# Antibiotic resistance breakers: current approaches and future directions

**DOI:** 10.1093/femsre/fuz014

**Published:** 2019-05-31

**Authors:** Mark Laws, Ali Shaaban, Khondaker Miraz Rahman

**Affiliations:** Institute of Pharmaceutical Sciences, School of Cancer and Pharmaceutical Sciences, King's College London, Franklin-Wilkins Building, 150 Stamford Street, London, SE1 9NH; Institute of Pharmaceutical Sciences, School of Cancer and Pharmaceutical Sciences, King's College London, Franklin-Wilkins Building, 150 Stamford Street, London, SE1 9NH; Institute of Pharmaceutical Sciences, School of Cancer and Pharmaceutical Sciences, King's College London, Franklin-Wilkins Building, 150 Stamford Street, London, SE1 9NH

**Keywords:** antibiotic resistance breakers, ESKAPEE, efflux pump inhibitors, membrane permeabilisers, beta-lactamase inhibitors, combination therapy

## Abstract

Infections of antibiotic-resistant pathogens pose an ever-increasing threat to mankind. The investigation of novel approaches for tackling the antimicrobial resistance crisis must be part of any global response to this problem if an untimely reversion to the pre-penicillin era of medicine is to be avoided. One such promising avenue of research involves so-called antibiotic resistance breakers (ARBs), capable of re-sensitising resistant bacteria to antibiotics. Although some ARBs have previously been employed in the clinical setting, such as the β-lactam inhibitors, we posit that the broader field of ARB research can yet yield a greater diversity of more effective therapeutic agents than have been previously achieved. This review introduces the area of ARB research, summarises the current state of ARB development with emphasis on the various major classes of ARBs currently being investigated and their modes of action, and offers a perspective on the future direction of the field.

## Teaser

This review explores the area of ARB research, summarises the current state of ARB development – including modifying enzyme inhibitors, membrane permeabilisers and efflux pump inhibitors – and offers a perspective on the future direction of the field.

## INTRODUCTION

Since their discovery more than 70 years ago, antibacterial drugs have become an essential part of the modern healthcare landscape, allowing treatment of previously life-threatening bacterial infections. However, ever-increasing levels of antimicrobial resistance (AMR) threaten the health benefits achieved with antibiotics and this phenomenon is recognised as a global crisis (Ventola 2015). Over the period of 2011–2014, the percentage of *Klebsiella pneumoniae* infections resistant to fluoroquinolones, third-generation cephalosporins or aminoglycosides, as well as combined resistance to all three antibiotic groups, has increased significantly in Europe, with a similar trend also observed for *Escherichia coli* infections (ECDC 2015). With AMR currently estimated to be responsible for 50 000 deaths annually across the US and Europe, urgent action needs to be taken on an international scale if the modern antibiotic treatment paradigm is to survive (O'Neill 2014). It should be noted that this review will discuss approaches to overcome bacterial resistance, but AMR refers to resistance caused by all microbes against their respective drugs.

While figures vary between different regions, the general trend is that poorer countries are experiencing much higher levels of resistance. This is likely due to several factors, including greater availability of second- and third-line treatments in ‘First World’ countries compared to their ‘Third World’ counterparts. Additionally, regional instances of higher resistance levels can have a global effect, with the advent of rapid intercontinental travel allowing the dissemination of resistant bacterial strains globally. It has been suggested that regional resistance levels could affect international travel and commerce, with people less likely to be willing to travel to areas where they could develop problematic bacterial infections. That AMR levels are only rising, despite implementation of additional healthcare measures in the more economically developed countries of the world, highlights the need for novel approaches to tackling the AMR problem (O'Neill 2014).

The effects of antibacterial resistance are not limited to those patients who develop bacterial infections; wider medical procedures stand to be impacted. Antibiotic prophylaxis is commonly employed to avoid the development of infections, both preoperatively for a variety of surgical procedures and for immunocompromised patients undergoing chemotherapy (Wenzel 1992; Teillant *et al*. 2015; Crader and Bhimji 2018). Such prophylactic measures will no longer be possible if AMR spreads at its current rate, which could in turn impact the scope of surgical procedures available to clinicians and the quality of patients’ lives (O'Neill 2014.).

The ESKAPE pathogens (*Enterococcus faecium*, *Staphylococcus aureus*, *Klebsiella pneumoniae, Acinetobacter baumannii*, *Pseudomonas aeruginosa* and *Enterobacter species*), whilst not the only problematic pathogens, have been identified as requiring special attention since they are responsible for the majority of hospital-acquired infections per annum and show high incidences of AMR (Rice 2008). With recent observations of strains of Gram-negative ESKAPE bacteria possessing multiple mechanisms of resistance to carbapenems, the drugs of last resort used to treat such infections, the need for new classes of antibiotics with novel modes of action is greater than ever (Limansky *et al*. 2002; Mena *et al*. 2006; Rodriguez-Martinez, Poirel and Nordmann 2009; Papp-Wallace *et al*. 2011). However, since the 1960s only two new antibiotic classes have been released and the scientific community has been unable to keep pace with the emergence of resistance (Coates, Halls and Hu 2011).

Investment in antibiotic research by major pharmaceutical companies has declined sharply in recent years, mainly because of the lack of return in investments. Besides a long and difficult regulatory process for new drugs to navigate (Ventola 2015), antibiotics are typically short term treatments meaning such drugs bring in less revenue for pharmaceutical companies when compared to drugs that are intended to treat long term conditions. In addition, the rise of other infectious diseases with different causative agents, such as acquired immune deficiency syndrome, has caused a shift in focus within the industry, often resulting in reduced budgets available for antibiotics research and development (Alanis 2005). With healthcare policy increasingly inclined towards the saving of new antimicrobials for treatment of resistant infections, change is required to make antibiotic development more attractive. Solutions include research incentives, such as the Innovative Medicines Initiative ‘New Drugs for Bad Bugs’ workstream which funds antimicrobial discovery research between academics/small enterprises and large companies, simplification of the regulatory landscape, such as the proposed FDA rapid antibacterial approval pathway LPAD (Limited Population Antibacterial Drug), and re-evaluation of the prices of antibiotics in order to provide companies with a better return on their investments (Sukkar 2013). Until the implementation of such incentives results in an increased volume of new antibiotic candidates reaching and passing the clinical trial hurdle, interim strategies must be explored to preserve the current clinical arsenal of antibiotics.

## ANTIMICROBIAL RESISTANCE

**Figure 1.**
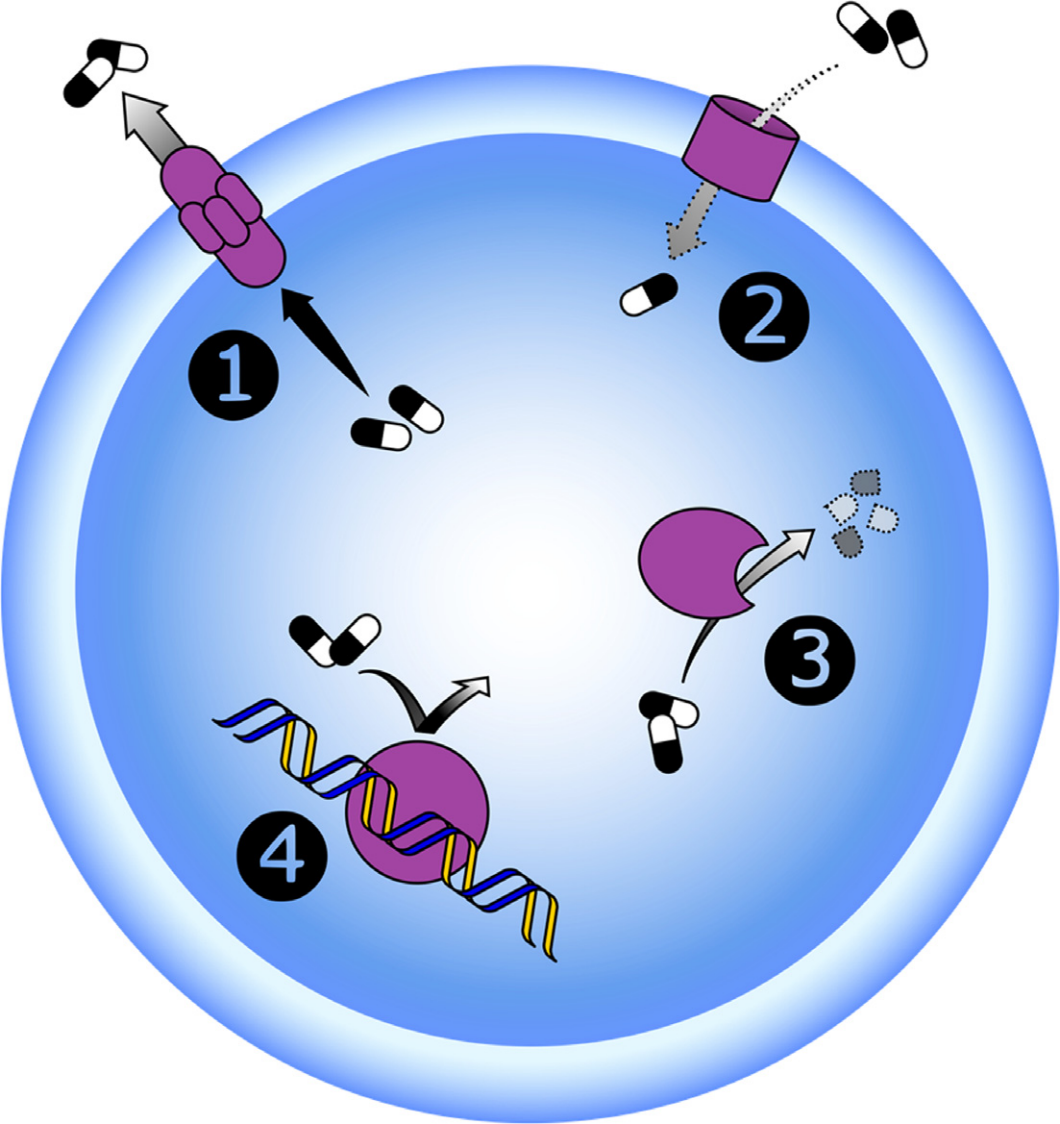
Bacterial resistance mechanisms to antibiotics. 1) Increased drug efflux; 2) decreased drug uptake; 3) drug modification/destruction and 4) target modification.

There are four main molecular mechanisms by which bacteria may resist the effects of antibiotics; modification of the target site, modification or destruction of the antibiotic, antibiotic efflux *via* efflux transporters and reduced antibiotic influx through decreased membrane permeability (Figure 1) (Munita and Arias 2016). These resistance mechanisms can be present together in different combinations in one bacterial cell, potentially allowing high level resistance to multiple antibiotic compounds simultaneously (Nikaido 2009). Some bacteria possess an innate insensitivity towards certain classes of antibiotics (intrinsic resistance), either through naturally possessing any of the above mechanisms in the absence of artificial antibacterial selection pressure (ampicillin resistance in *Klebsiella* spp.), lack of the antibiotic target (vancomycin resistance in lactobacilli) or lack of a metabolic pathway or enzyme necessary for the activation of the drug (metronidazole resistance in aerobic bacteria) (Bryan and Kwan 1981; Schaechter *et al*. 2007).

Resistance towards antibiotics is acquired by bacteria through either vertical evolution (endogenous) or horizontal evolution (exogenous). Vertical evolution involves the occurrence of a spontaneous mutation within the bacterial genome that confers on the bacterium (and subsequently its progeny) increased resistance to a given compound. The process to achieve high level resistance is often stepwise, wherein the selection pressure of antibiotic treatment causes an initial mutation that allows domination of the pathogen population by the mutant bacteria, followed by subsequent additional mutations that confer an additional survival advantage during further antibiotic therapy. Though mutation frequencies can often be as low as 10^−8^, this is offset by the vast numbers of cells in bacterial colonies (Drlica and Perlin 2011). Work by Santos Costa *et al*. into fluoroquinolone resistance in *S. aureus* showed that, in this case at least, an intermediate resistance phenotype (*via* upregulation of efflux pump expression) is first to appear and acts as a platform from which higher level resistance mutations can occur by ensuring a sub-lethal intracellular fluoroquinolone concentration (Santos Costa *et al*. 2015).

Horizontal evolution involves the transfer of a resistance gene from a resistant bacterium to a susceptible bacterium. The mechanisms through which it can occur are conjugation, transduction and transformation. Conjugation involves the transfer of resistance (R) plasmids containing antibiotic resistance genes between bacteria through a conjugative pilus, whilst transformation refers to the alteration of the bacterial genome through the uptake and incorporation of exogenous DNA and transduction involves transfer of bacterial DNA as facilitated by a viral vector. Such transfer mechanisms potentially allow a mechanism acquired by less problematic bacterial strains to spread to a more dangerous bacterial species, with potentially devastating consequences (Alanis 2005).

The genes encoding different resistance mechanisms are often located on transposons, which makes it easier for them to be transmitted between different bacteria, and some transposons may contain specialised regions called integrons able to include different resistant genes, thereby making a bacterial species resistant to multiple different antibiotics (Alanis 2005). In addition, bacteria can also have physical states which aid in resisting antibacterial pressure. A variety of both Gram-positive and Gram-negative bacterial species are known assemble in biofilms (Abee *et al*. 2011), hydrated matrices of extracellular polymeric substance in which the bacterial cells are embedded allowing adherence to both each other and external surfaces. Such structures become problematic when located in urinary catheters or on medical implants; since biofilms are harder for antibiotics to penetrate at lethal concentrations, the biofilm provides resistance to antibiotic action (Donlan 2002). The bacterial population within the biofilm can also enter into a dormant state where they are not actively growing, and this can also contribute to antibiotic resistance (Gilbert, Collier and Brown 1990; Wood, Knabel and Kwan 2013).

## ANTIBIOTIC RESISTANCE BREAKERS

To tackle the increasing emergence of AMR, alternative treatment strategies have been designed with the collective aim of reducing the number of antibiotics used and preserving the current classes of antibiotic for further clinical use. This review aims to showcase the potential of one such strategy, the use of antibiotic resistance breakers (ARBs). These are compounds that can increase the effectiveness of current antibiotics by combatting the resistance mechanisms employed against them. ARBs may or may not have direct antibacterial effects and can either be co-administered with or conjugated to failing antibiotics. Though ARBs have previously been referred to as antibiotic adjuvants, the latter also refers to alternative treatments such as drugs which stimulate host defence mechanisms to aid the eradication of bacterial infections (Gill, Franco and Hancock 2015); as such, this review will be restricted to the discussion of compounds that are used to reverse bacterial resistance mechanisms. The major classes of ARBs currently under investigation include modifying-enzyme inhibitors, membrane permeabilisers and efflux pump inhibitors (EPIs).

The idea of co-administering ARBs with conventional antibiotics stems from dual antibiotic therapy, which has enjoyed success in the past through either synergistic or additive effects of the individual antibiotic agents (Kalan and Wright 2011), and several ARBs have enjoyed lengthy clinical use including the β-lactamase inhibitors (BLIs) (Drawz and Bonomo 2010). Successful co-administered ARBs should enhance the effects of antibiotics by combatting the bacterial resistance mechanisms employed against the latter, allowing lower doses of antibiotics to be used. The minimum inhibitory concentration (MIC), the minimal concentration required of a compound to prevent visible growth of the pathogenic species under defined conditions (Wiegand, Hilpert and Hancock 2008), is a useful term in this regard; the more successful ARBs achieve greater reductions in the MICs of antibiotics versus antibiotic monotherapy. Such potentiation is an attractive prospect, both because reduced antibiotic selection pressure could slow the onset of resistance and because widening of the therapeutic window may allow for the alleviation of side effects experienced by patients on antibiotic monotherapy.

### Modifying enzyme inhibitors

Bacteria employ a diverse range of enzymes to modify or destroy antibiotics in order to render them ineffective and achieve a resistant phenotype. These enzymes can be categorised by both their mechanisms of action and their substrate antibiotics. Hydrolysis of certain susceptible bonds within the antibiotic molecule, transfer of a functional group to the antibiotic and (less commonly) the actions of redox and lyase enzymes are all examples of detoxification mechanisms (Wright 2005). This led to the development of antibiotics that would tolerate their actions, such as the β-lactam flucloxacillin which was designed to tolerate the action of the penicillinases (Sutherland, Croydon and Rolinson 1970). A method which has found more success is the design of modifying enzyme inhibitors, a term which encompasses the wide variety of chemical compounds that target bacterial enzymes involved in antibiotic modification and destruction. Modifying enzyme inhibitors are used to disrupt bacterial detoxification enzymes, increasing the effectiveness of a co-administered antibiotic. Two major classes are the BLIs and aminoglycoside-modifying enzymes.

#### B-lactamase inhibitors

The most successful class of ARBs is arguably the BLIs. β-lactam antibiotics function by interfering with bacterial cell-wall synthesis, binding to and inactivating the C-terminal transpeptidase domain of penicillin-binding proteins which are responsible for the cross-linking of the peptidoglycan chains in the cell wall (Fisher *et al*. 2005). The β-lactams include several frequently prescribed families of antibiotics such as the penicillins and cephalosporins. They remain the most widely used class of antibiotics, reported to comprise 65% of the global antibiotic market in 2004 (Elander 2003), while broad-spectrum penicillins and cephalosporins were reported to be the two most consumed drug classes globally in 2010 (Van Boeckel *et al*. 2014). β-lactamases (EC 3.5.2.6) are bacterial enzymes that hydrolyse the β-lactam rings such drugs possess, inactivating them. Modification of β-lactam drugs is the major defence mechanism for Gram-negative pathogenic bacteria, with β-lactamases differing in their mechanisms and their substrate specificities (Wilke, Lovering and Strynadka 2005). Of note, carbapenemases can often act on carbapenem drugs and a wide range of other β-lactams, including penicillins, cephalosporins and monobactams (Queenan and Bush 2007). These enzymes are of special concern, since carbapenems are generally reserved as a last resort for many complicated infections, including those caused by both Gram-positive and Gram-negative bacteria (Papp-Wallace *et al*. 2011).

There are two widely accepted classification systems for β-lactamases. The Ambler molecular classification divides them into classes A-D based on sequence homology, each of which function *via* slightly different mechanisms. All four classes hydrolyse the β-lactam ring, but enzymes of classes A, C and D do so through use of a serine nucleophile, whereas those of class B require a metal cofactor, usually a zinc atom, to achieve the same effect. Because of the need for the metal cofactor, class B β-lactamases may also be referred to as metallo-β-lactamases (MBLs) (Ambler 1980). An alternative classification, known as the Bush-Jacoby-Medeiros functional classification, is based on substrate specificity and includes four main groups based on inhibitor profile, with group 2 further divided into several subgroups (Bush and Jacoby 2010). The extended spectrum β-lactamases (ESBLs), often loosely defined as β-lactamases which confer resistance against penicillins, aztreonam and first, second and third generation cephalosporins, are recognised as particularly problematic. ESBLs may be regarded as members of class A of the Ambler molecular classification; with the OXA-type β-lactamases being an exception, named after their ability to hydrolyse oxacillin and members of class D. Carbapenems are usually regarded as the drugs of choice to eradicate strains possessing ESBLs. However, several ESBL-producing clinical isolates have been identified which are resistant to carbapenems (Paterson and Bonomo 2005). For example, a *P. aeruginosa* strain has been identified which produces both the ESBL PER-1 and the carbapenemase VIM-2 (Docquier *et al*. 2001).

**Figure 2.**
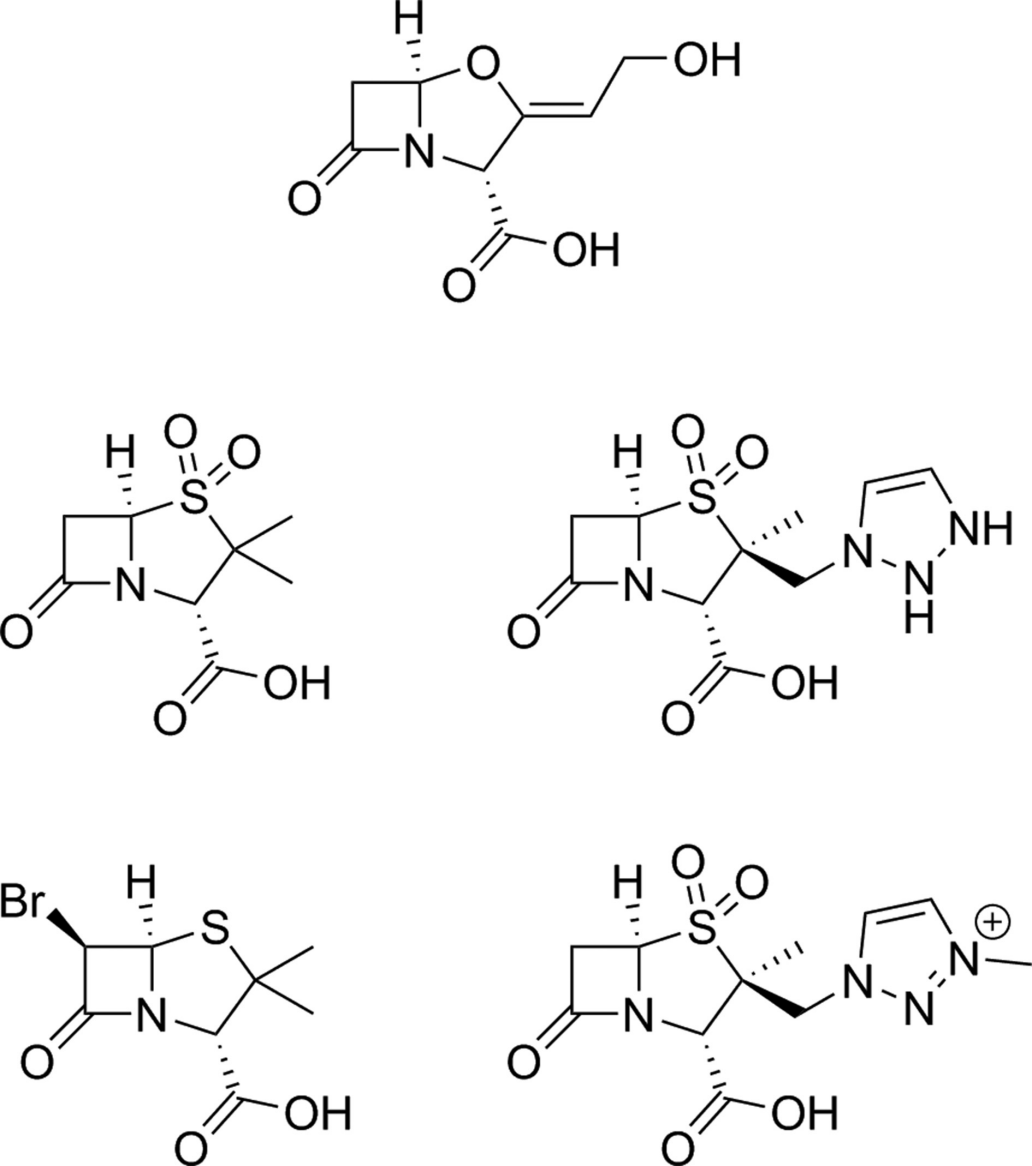
Classic BLIs. Structures of clavulanic acid (top), sulbactam (middle left), tazobactam (middle right), brobactam (bottom left) and AAI101 (bottom right) (English *et al*. 1978; Reading, Farmer and Cole 1983; Aronoff *et al*. 1984; Wise *et al*. 1992; Mushtaq *et al*. 2014; Nordmann *et al*. 2014).

Several BLIs widely used in the clinical setting are themselves β-lactam compounds. One well documented example is clavulanic acid (Fig. 2), commonly sold as the combination products co-amoxiclav (combined with the β-lactam amoxicillin; marketed by GlaxoSmithKline as Augmentin®) and co-ticarclav (combined with ticarcillin; marketed by GlaxoSmithKline as Timentin®). While clavulanic acid displays poor antimicrobial activity *in vivo* (Reading, Farmer and Cole 1983), its β-lactamase inhibitory activity affords the co-administered β-lactam protection from enzymatic degradation (Bush 1988). Predominantly active against Ambler class A β-lactamases, clavulanic acid irreversibly acylates the catalytic serine residue, resulting in an inactive acyl-enzyme complex. Clavulanic acid has been shown to inhibit the plasmid-encoded β-lactamases of *E. coli* and *S. aureus*, but not the chromosomally-encoded versions found in *Pseudomonas* and *Enterobacter* strains (Wright 1999). Thus, co-amoxiclav has activity against both amoxicillin-sensitive and select amoxicillin–resistant strains of clinically-relevant pathogenic microorganisms. However, Leflon-Guibout *et al*. studied co-amoxiclav resistance in *E. coli* clinical isolates, defined by an MIC greater than 16 μg mL^−1^, in 14 French hospitals from 1996 to 1998 and found that the overall resistance rate was 5% with most resistant isolates identified in patients with respiratory tract infections (Leflon-Guibout *et al*. 2000). Such reports of resistance to the established BLIs (Drawz and Bonomo 2010) have driven fresh efforts into finding novel alternatives.

Sulbactam (CP-45,899; developed by Pfizer (English *et al*. 1978)) and tazobactam (YTR 830 H; developed by Taiho Pharmaceutical Co. (Aronoff *et al*. 1984)) are both penicillanic acid sulfones with β-lactamase inhibitory activity (Fig. 2). Both compounds inhibit TEM-type β-lactamases (IC_50_s of 0.03 and 0.01 against TEM-3, respectively), though sulbactam is far less effective against SHV- and OXA-type β-lactamases (Payne *et al*. 1994). Available combinations of sulbactam with β-lactam antibiotics include ampicillin-sulbactam, which shows limited activity against ESBL-producers including strains of *E. coli* and *K. pneumoniae* (Rafailidis, Ioannidou and Falagas 2007), and cefoperazone-sulbactam, effective against ESBL-positive strains of *Pseudomonas* spp., *Acinetobacter* spp., *Klebsiella* spp. and *E. coli* (Bodey, Miller and Ho 1989; Mohanty *et al*. 2005). Sulbactam has also been shown to inhibit penicillin binding protein 3 in *Acinetobacter* spp., granting it direct antibacterial activity against this genus (Penwell *et al*. 2015).

Combinations of β-lactams and tazobactam currently in clinical use include ceftolozane-tazobactam and piperacillin-tazobactam. Ceftolozane-tazobactam was approved in December 2014 by the FDA for treatment of complicated intra-abdominal and urinary tract infections and shows activity against multidrug-resistant (MDR) *P. aeruginosa* (MIC_50_ 2 μg mL^−1^; MIC_90_ 8 μg mL^−1^), ESBL-negative *K. pneumoniae* (MIC_50_ 0.25 μg mL^−1^; MIC_90_ 0.5 μg mL^−1^) and ESBL-positive *E. coli* (MIC_50_ 0.5 μg mL^−1^; MIC_90_ 4 μg mL^−1^), among others. A dose reduction is required for patients with renal impairment, depending on creatinine clearance (Cho, Fiorenza and Estrada 2015). In comparison, work by Mohanty *et al*. demonstrated the piperacillin-tazobactam combination, approved by the FDA in 1993 (Shlaes 2013), to have superior percentage coverage of ESBL-positive strains of *Pseudomonas* spp., *Klebsiella* spp., *E. coli*, *Enterobacter* spp. and *Citrobacter* spp. versus cefoperazone-sulbactam and ticarcillin-clavulanic acid (Mohanty *et al*. 2005). In India, the cephalosporin cefepime has been used with tazobactam and this combination is gaining ground as an attractive new prospect for the treatment of MDR Gram-negative pathogens (Bush 2015; Livermore *et al*. 2018).

Brobactam (BRL 25 214; Fig. 2), structurally similar to sulbactam and tazobactam, was developed by LEO Pharma A/S as another BLI (Wise *et al*. 1992). The work of Melchior and Keilding demonstrated that brobactam alone possessed 8–50 fold higher potency than clavulanic acid against chromosomally-encoded cephalosporinase enzymes in Enterobacteriaceae and that an ampicillin-brobactam combination held superior activity *in vitro* to co-amoxiclav against *Proteus vulgaris*, *Morganella morganii*, *Citrobacter freundii* and *Yersinia enterocolitica* (Melchior and Keiding 1991). However, despite favourable results for a combination of brobactam and the β-lactam prodrug pivampicillin from an eight-person tolerability study (Wise *et al*. 1992), development of brobactam appears to have been discontinued and it is not available for use in the clinic.

AAI101 (Fig. 2), a novel penicillanic acid sulfone similar in structure to tazobactam, is an ESBL inhibitor active against some class A and D carbapenemases (Mushtaq*et al*. 2014; Nordmann *et al*. 2014) that is being developed by Allecra Therapeutics as a combination therapy with cefepime. Crandon and Nicolau reported that the combination (using 8 μg mL^−1^ of AAI101) was effective against a panel of 223 cefepime-resistant Enterobacteriaceae isolates, improving on the MIC_50_ of cefepime by over 512-fold (Crandon and Nicolau 2014; Crandon and Nicolau 2015). They subsequently demonstrated a strong correlation between increasing AAI101 concentration and MICs for the cefepime-AAI101 combination in *K. pneumoniae*-infected female ICR mice between 1 and 16 μg mL^−1^ (Crandon and Nicolau 2015). As of 2017, the combination is in phase II clinical trials (Papp-Wallace *et al*. 2017).

**Figure 3.**
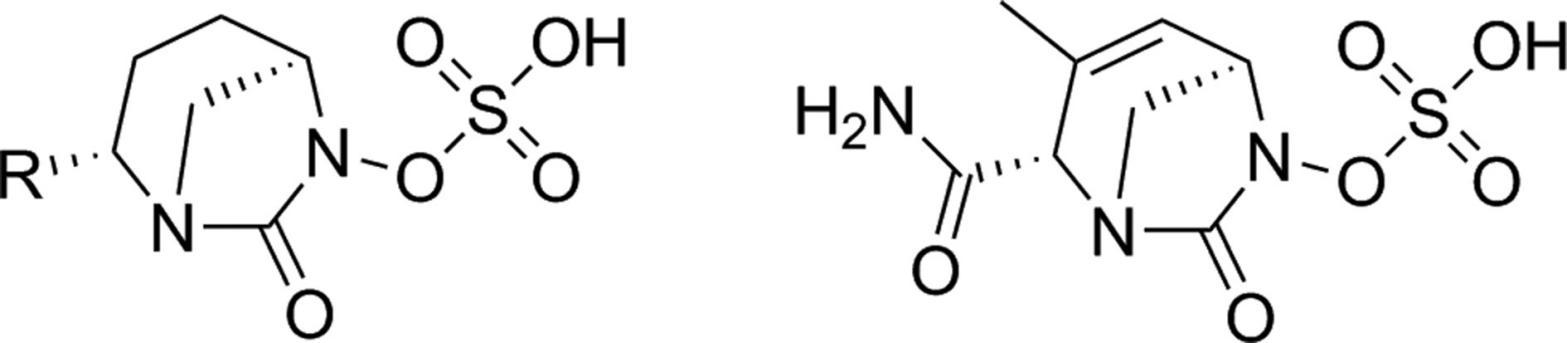
The DABCOs. General structure of DABCOs (left) and structure of ETX2514 (right) (Mangion *et al*. 2011; Durand-Reville *et al*. 2017).

As early as the late 1980s, nosocomial isolates resistant to combination therapies involving the aforementioned β-lactam-based BLIs were being reported (Legrand *et al*. 1988; Ling *et al*. 1988; Eliopoulos *et al*. 1989; Cullmann and Stieglitz 1990). New BLIs were required for the next generation of combination treatments, and to this end classes of structurally divergent compounds with BLI activity were investigated. One such class of newer, non-β-lactam BLIs is the diazabicyclooctanes (DABCOs), based on a (5R)-7-oxo-1,6-diazabicyclo[3.2.1]octan-6-yl sulphate core (Fig. 3). The strained nature of this core, further activated towards nucleophilic attack through the incorporation of a sulphate group on one nitrogen of the urea functionality, underlies the β-lactamase inhibitory activities of the DABCOs (Mangion *et al*. 2011). Fig. 3 and Table 1 list the structures of the compounds in this class either approved for clinical use or currently in development.

**Table 1.** Structures of DABCOs currently in clinical use/in development (Mangion *et al*. 2011; Maiti *et al*. 2013; Garber 2015; Patil *et al*. 2016; Bush and Page 2017; Papp-Wallace *et al*. 2018; Thye 2018).

Name	R group	Name	R group
Bicyclic urea core	H	Zidebactam	
			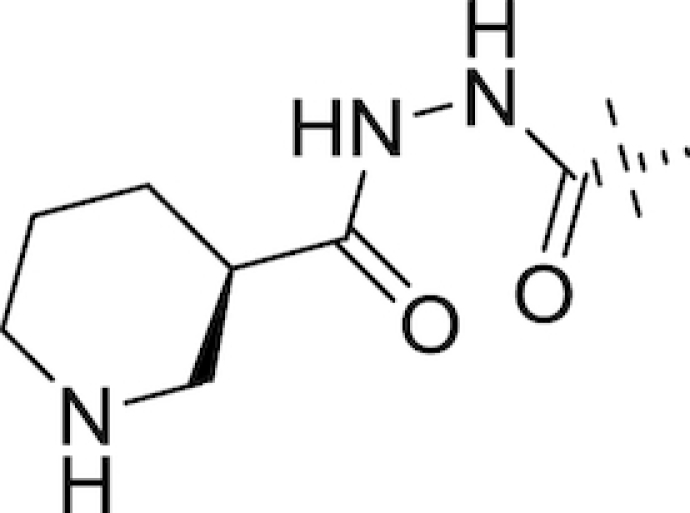
Avibactam	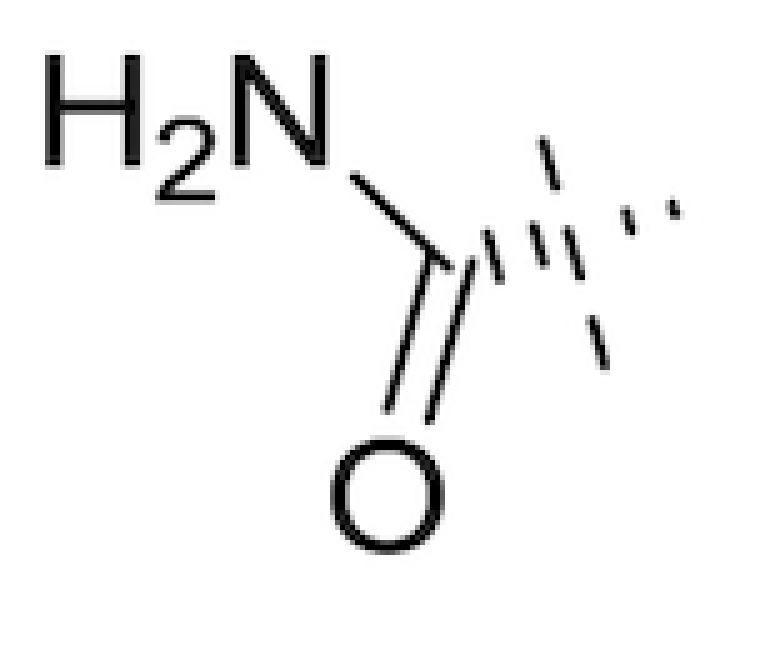	WCK 5153	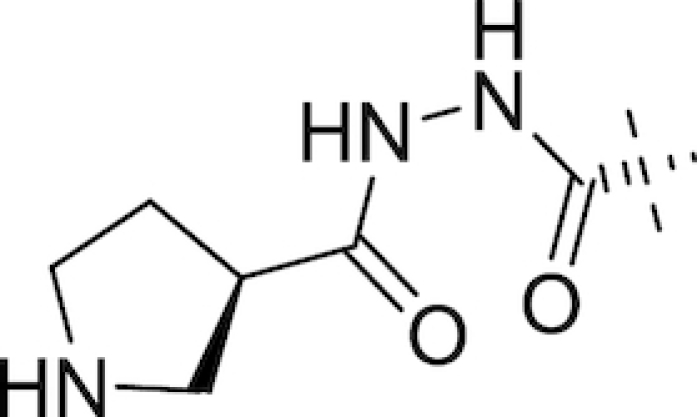
Relebactam	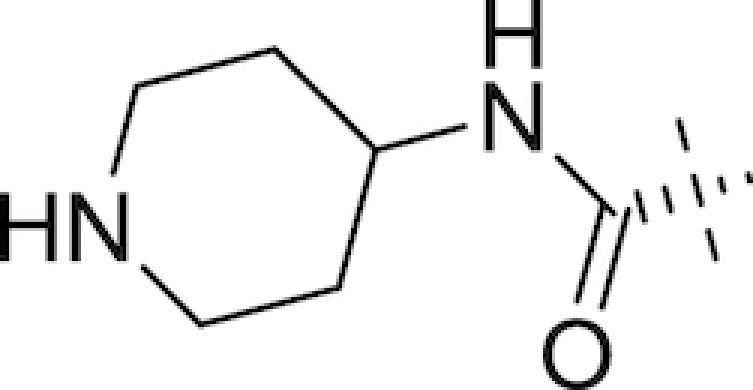	WCK 4234	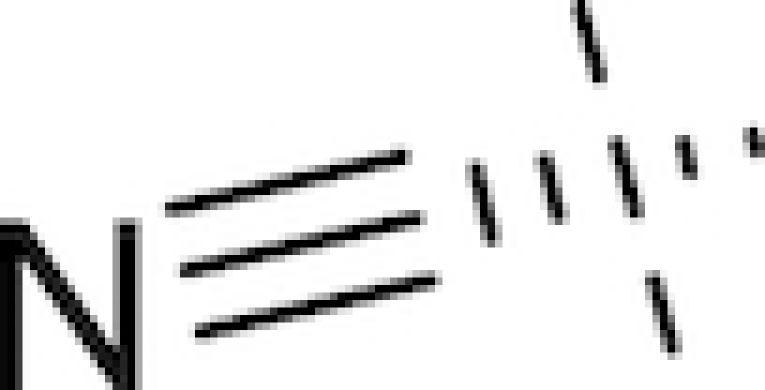
Nacubactam	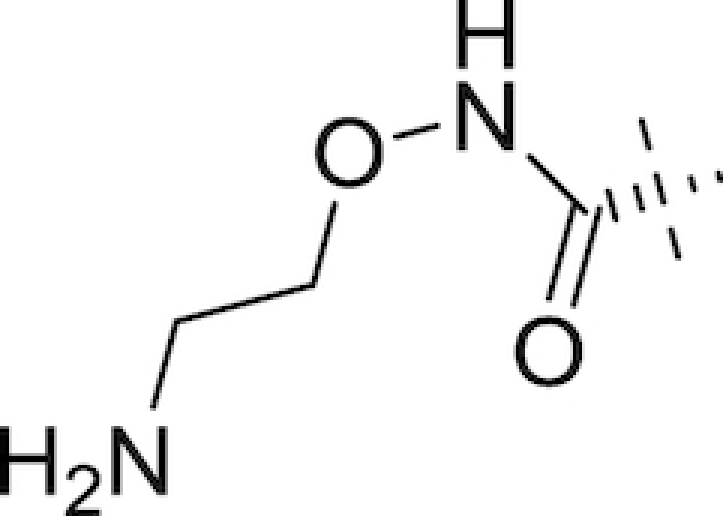	GT-055	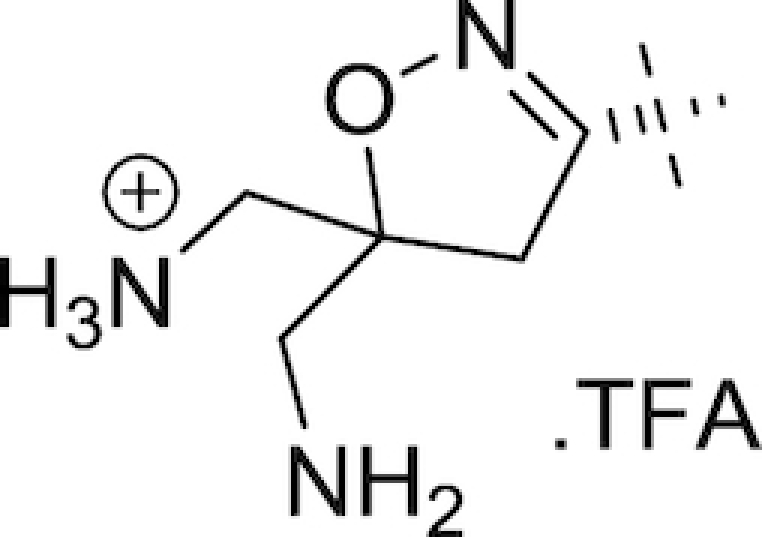

Avibactam (NXL104; developed by Actavis and AstraZeneca; Table 1), when approved for clinical use in the US in 2015, was both the first DABCO brought to market and the first new BLI approved in 22 years (Garber 2015). A comparison by Stachyra *et al*. of avibactam with clavulanic acid, sulbactam and tazobactam showed the former to be superior in inhibitory activity for all Ambler class A and C β-lactamases tested, including TEM-1, KPC-2 and SHV-4; investigation of the mode of action revealed that avibactam covalently modifies a catalytic serine residue in the β-lactamase active site in the same manner as the penicillanic sulfones, but that the highly stable nature of the carbamyl-enzyme complex underpins its enhanced inhibitory activity (Stachyra *et al*. 2010). In combination with the third-generation cephalosporin ceftazidime (marketed as Avycaz® in the US by Allergan and as Zavicefta® in Europe by Pfizer), it is approved by the FDA for the treatment of complicated intra-abdominal infections in combination with metronidazole and for the treatment of complicated urinary tract infections (Mosley *et al*. 2016; Wright 2016). The combination has broad Gram-negative activity, including Enterobacteriaceae and *P. aeruginosa* (Crandon *et al*. 2012; Flamm *et al*. 2014; Chalhoub *et al*. 2015; Sader *et al*. 2015), and was found to be superior to ceftazidime alone against 120 KPC-producing carbapenem-resistant Enterobacteriaceae clinical isolates in a study by Castanheira and co-workers in 2015 (Castanheira *et al*. 2015). However, while a subsequent study by Castanheira *et al*. found 99.3% of Enterobacteriaceae isolates from US hospitals between 2012 and 2015 were be susceptible to ceftazidime-avibactam (Castanheira *et al*. 2017a), Shields *et al*. have since detailed the first instances of *K. pneumoniae* ceftazidime-avibactam resistance in patients treated with the combination for 10–19 days. They identified the causes of said resistance to be mutations (chiefly a D179Y/T243M double mutation) in the plasmid-located *bla*_KPC-3_ gene (Shields *et al*. 2017). As with ceftolozane-tazobactam, patients with renal impairment require a dose reduction according to creatinine clearance levels (Mosley *et al*. 2016). The separate combinations of avibactam and both ceftaroline and aztreonam are in late-stage clinical trials (ClinicalTrials.gov, NCT03329092) (Wright 2016).

Another DABCO in late stage development is relebactam (MK-7655; Merck & Company, Inc.; Table 1), currently being investigated for combination with the carbapenem imipenem and the dehydropeptidase I inhibitor cilastatin (the latter employed to prevent degradation of imipenem in the kidneys). The structure of relebactam is similar to that of avibactam, with a piperazine ring added to the nitrogen of the C2 amide substituent (Mangion *et al*. 2011). Zhanel *et al*. have recently compiled a series of modal MIC_50_ and MIC_90_ values for both imipenem and imipenem-relebactam based on a review of available *in vitro* studies; they report that relebactam enhances the activity of imipenem versus the majority of Enterobacteriaceae, including KPC-producing *K. pneumoniae* (>16 fold reduction in MIC_90_), and imipenem-resistant *P. aeruginosa* (8-fold reduction), though no enhancement was observed against KPC-producing *P. aeruginosa* or *A. baumannii* (Zhanel *et al*. 2018). As of September 2017, Merck have completed a phase III trial for evaluation of the safety and efficacy of the imipenem-cilastatin-relebactam combination versus imipenem-cilastatin-colistimethate sodium in treatment of imipenem-resistant bacterial infections (ClinicalTrials.gov, NCT02452047). A number of clinical trials are currently underway involving imipenem-cilastatin-relebactam, including a phase III trial in Japan investigating treatment of complicated intra-abdominal infections and complicated urinary tract infections (ClinicalTrials.gov, NCT03293485), a phase III noninferiority trial versus piperacillin-tazobactam for treatment of hospital-acquired and ventilator-associated bacterial pneumonia (ClinicalTrials.gov, NCT02493764) and a phase I trial investigating the individual pharmacokinetic profiles of the three drugs following administration (ClinicalTrials.gov, NCT03230916).

In August 2013, Naeja Pharmaceutical Inc. were granted a patent for a promising series of novel C2 N-(hydroxy)amide and hydrazide DABCOs (Maiti *et al*. 2013), subsequently in-licensed to Fedora Pharmaceuticals. Lead compounds identified within this series included FPI-1459, FPI-1465, FPI-1523 and FPI-1602 and, unlike previous DABCOs, they were found to have multiple modes of action; in addition to their BLI activities, they act directly as antibacterial agents through inhibiting penicillin-binding protein 2 and also as potentiators of their accompanying antibiotics in the absence of β-lactamases (Morinaka *et al*. 2015; King *et al*. 2016; Bush and Page 2017; Bush 2018). Meiji Seika Pharma and Fedora Pharmaceuticals partnered with F. Hoffmann la Roche in January 2015 (Philippidis 2015) to further develop FPI-1459, renamed nacubactam (RO7079901, previously OP0595, RG6080). In separate papers, Morinaka and co-workers investigated the ability of nacubactam to resensitise CTX-M-15 positive *E. coli*, KPC-positive *K. pneumoniae* and AmpC-derepressed *P. aeruginosa* to piperacillin, meropenem and cefepime both *in vitro* and *in vivo*; they found 4 μg mL^−1^ of nacubactam sufficient to achieve mean MICs of <0.03 *in vitro* for all three β-lactams in the *E. coli* and *K. pneumoniae* strains tested (Morinaka *et al*. 2016). At this concentration, only the cefepime combination achieved a mean MIC below 2 μg mL^−1^ in the *P. aeruginosa* strains tested, indicating cefepime to be the optimal β-lactam partner for nacubactam (Morinaka *et al*. 2017). Nacubactam was also observed to retain its β-lactam enhancer effect in Enterobacteriaceae possessing nacubactam-resistant MBLs (Livermore *et al*. 2016a). Having completed a phase I trial in 2014 to assess safety and tolerability in adult Caucasian males (ClinicalTrials.gov, NCT02134834), nacubactam (Table 1) has since been involved in a number of other phase I trials and appears to be in development in combination with meropenem for the treatment of meropenem-resistant Gram-negative infections (ClinicalTrials.gov, NCT03182504, NCT03174795, NCT02972255 & NCT02975388).

Previously known as WCK 5107, zidebactam (Table 1) is in development by Wockhardt Ltd (Bush and Page 2017) and was originally patented in 2013 (Patel *et al*. 2013). Zidebactam inhibits class A, C and select class D β-lactamases (Khande *et al*. 2016) and, like nacubactam, possesses both direct activity against MDR Gram-negative bacteria (Deshpande *et al*. 2016) and the capability to augment the activity of β-lactams in the absence of β-lactamases (Livermore *et al*. 2016b) in a number of species including *A. baumannii* (Moya *et al*. 2017a) and *P. aeruginosa* (Moya *et al*. 2017b). A combination with cefepime, also known as WCK 5222 and FED-ZID, was shown to be effective *in vitro* against a global collection of 7876 clinical isolates from 2015, consisting of Enterobacteriaceae, *P. aeruginosa* and *Acinetobacter* spp.; in a 1:1 ratio, the combination achieved MICs below or equal to 4 μg mL^−1^ in 99.9% of Enterobacteriaceae isolates and MICs below or equal to 8 μg mL^−1^ in 99.5% of *P. aeruginosa* isolates (Sader *et al*. 2017a). These results are in concurrence with a subsequent, smaller scale study conducted by Sader and co-workers (Sader *et al*. 2017b). However, a separate study by Livermore and co-workers found cefepime-zidebactam to be ineffective (over 32 μg mL^−1^) against Proteeae, *Serratia* spp. and select strains of *E. coli*, *Klebsiella* spp., *Enterobacter* spp. and *Citrobacter* spp. (Livermore *et al*. 2017). A number of phase I clinical trials have been completed investigating the safety, tolerability and pharmacokinetics of zidebactam, both alone (ClinicalTrials.gov, NCT02674347) and in combination with cefepime (ClinicalTrials.gov, NCT02532140 & NCT02707107) (Preston *et al*. 2019).

Also in development by Wockhardt Ltd is WCK 5153 (Table 1), a close structural analogue of WCK 5107 differing only in the nature of the aliphatic ring of the side arm moiety (a 3-subsituted piperidine in WCK 5107, a 3-subsituted pyrrolidine in WCK 5153). WCK 5153 inhibits class A, C and some class D β-lactamases, with increased potency versus class C enzymes compared to both avibactam and relebactam (Papp-Wallace *et al*. 2018). As for WCK 5107, WCK 5153 shows a β-lactam enhancer effect against *A. baumannii* (Moya *et al*. 2017a) and *P. aeruginosa* (Moya *et al*. 2017b), with the combination of cefepime and WCK 5107 achieving MICs of 0.06–4 μg mL^−1^ in a panel of *P. aeruginosa* strains including porin mutants (Moya *et al*. 2017b).

Another DABCO in development by Wockhardt Ltd. is WCK 4234 (Table 1). Like zidebactam, it is active against class A, C and some class D β-lactamases (Patil *et al*. 2016). A combination with meropenem, known as WCK 5999, has been shown to be superior to meropenem monotherapy against MDR clinical isolates of *A. baumannii* (Huband *et al*. 2016), including OXA-23- and OXA-24-producing strains (Castanheira *et al*. 2016a; Mushtaq *et al*. 2017), *K. pneumoniae* (Castanheira *et al*. 2016b) and *P. aeruginosa* (Huband *et al*. 2016).

An effort by Entasis Therapeutics towards the rational design of analogues of avibactam with improved Gram-negative penetration and better activity against class D β-lactamases led to the discovery of ETX2514 (Fig. 3), a DABCO analogue with class A, C and broad class D β-lactamase inhibitory activity (Durand-Reville *et al*. 2017). In particular, activity against the class D enzymes OXA-10, OXA-23 and OXA-24 is significantly improved in ETX2514 versus avibactam (Shapiro *et al*. 2017). Rate constants of time-dependent β-lactamase inhibition for ETX2514 (compared to avibactam) were approximately 100-fold higher for class A and C enzymes and approximately 1000-fold higher for class D β-lactamases. ETX2514 was also observed to be an inhibitor of penicillin binding protein 2 in *E. coli* and *A. baumannii*, helping explain its direct antibacterial activity against wider Enterobacteriaceae including *mcr-1*-positive *E. coli* (MIC_90_ 1 μg mL^−1^, 10 strains), *K. pneumoniae* (MIC_90_ 4 μg mL^−1^, 20 strains), *Enterobacter cloacae* (MIC_90_ 1 μg mL^−1^, 10 strains), *Stenotrophomonas maltophilia* (MIC_90_ 16 μg mL^−1^, 18 strains), *Citrobacter* spp. (MIC_90_ 2 μg mL^−1^, 55 strains) and class B β-lactamase-positive and -negative CRE (MIC_90_ 8 μg mL^−1^, 32 strains). The compound was well tolerated up to 2 g kg^−1^ in both rats and dogs in separate 14-day toxicological studies (Durand-Reville *et al*. 2017).

Imipenem, meropenem, ceftazidime and aztreonam were combined individually with ETX2514 (4 μg mL^−1^) against panels of 202 random *E. coli* clinical isolates and 202 random *P. aeruginosa* clinical isolates, respectively; MIC_90_ values for all four combinations against the *E. coli* panel were below 0.06 μg mL^−1^, whereas imipenem was the most effective β-lactam partner versus *P. aeruginosa* (MIC_90_ 2 μg mL^−1^). In a similar manner, the same four β-lactams and sulbactam were trialled with ETX2514 against 198 random *K. pneumoniae* clinical isolates; in this case, sulbactam was the most effective partner (MIC_90_ 4 μg mL^−1^). The sulbactam-ETX2514 combination was further tested against 1131 *A. baumannii* clinical isolates, including MDR, meropenem-resistant and colistin-resistant phenotypes, and improved upon sulbactam monotherapy 16-fold (64–4 μg mL^−1^) (Durand-Reville *et al*. 2017). McLeod *et al*. investigated the frequency of spontaneous resistance to sulbactam-ETX2514 in four different *A. baumannii* clinical isolates and found it to be low (< 9.0 × 10^−10^ frequency at 4x MIC of combination) with no ETX2514-resistant β-lactamases detected in resistant mutants (McLeod *et al*. 2018). ETX2514 has completed an open-label, phase I study (Rodvold *et al*. 2018) in 30 healthy adults in combination with sulbactam and a 124-person trial both alone and in combination with sulbactam compared with imipenem/cilastatin and a placebo to evaluate its safety, tolerability and pharmacokinetic profile (ClinicalTrials.gov, NCT02971423). As of April 2018, the combination is undergoing a phase I trial in 30 patients with varying degrees of renal impairment (ClinicalTrials.gov, NCT03310463) and phase II trial in 80 patients with complicated urinary tract infections (ClinicalTrials.gov, NCT03445195).

Also in development by Entasis Therapeutics is the DABCO ETX0282, an oral prodrug of ETX1317 (structures not yet disclosed). ETX1317, in line with other members of this class of BLI, enjoys activity against class A, C and D β-lactamases. Like ETX2514, it is an inhibitor of penicillin binding protein 2 in *E. coli*, affording the compound intrinsic antimicrobial activity. Based on testing in combination against a SHV-18, OXA-2 and OKP-6 positive strain of *K. pneumoniae*, cefpodoxime was selected as the optimal partner for ETX1317 at 4 μg mL^−1^ of the latter (Durand-Réville 2017). Pharmacokinetic studies in both rats and dogs showed both the prodrug and active form to have high bioavailabilities (>90%) and similar elimination half-lives to cefpodoxime. The combination of ETX0282 and cefpodoxime was effective at the three concentrations of BLI tested (10, 25 and 100 mg kg^−1^; cefpodoxime proxetil fixed at 50 mg kg^−1^) against *E. coli* ARC2687 in a neutropenic murine thigh infection model and against CRE *K. pneumoniae* ARC5118 at 200 and 400 mg kg^−1^ BLI concentrations (O'Donnell *et al*. 2017). In addition, the combination of cefpodoxime and 4 μg mL^−1^ ETX1317 was active against 33 of a 35 isolate panel of KPC and/or MBL-positive Enterobacteriaceae (MICs < 0.5 μg mL^−1^), outperforming ceftazidime and 4 μg mL^−1^ avibactam (McLeod *et al*. 2017). ETX0282 is currently undergoing a phase I clinical trial in healthy volunteers to evaluate pharmacokinetics and safety (ClinicalTrials.gov, NCT03491748).

The DABCO GT-055 is another promising BLI in development. Originally developed by LegoChem Biosciences (South Korea) as LCB18 0055 and subsequently licensed to Geom Therapeutics (Table   1), GT-055 is being developed in combination with the novel siderophore-conjugated cephalosporin GT-1 (also developed by LegoChem Biosciences and subsequently licensed to Geom Therapeutics). GT-055 is active against class A, C, D and some class B β-lactamases, has intrinsic activity against some Enterobacteriaceae and is reported to potentiate GT-1 against MDR strains of *A. baumannii* and *P. aeruginosa* (Thye 2018). Against a panel of 334 Enterobacteriaceae clinical isolates, GT-055 was observed to increase GT-1 activity against MBL-positive *E. coli* and *K. pneumoniae* strains (MIC_50/90_ 4/8 μg mL^−1^ for both), including porin/efflux mutant strains (16-fold lower MIC_90_, from 64 μg mL^−1^ to 4 μg mL^−1^) (Sader *et al*. 2018). The combination was found to improve upon GT-1 alone in a *K. pneumoniae* murine infection model (Oh *et al*. 2018). The combination showed activity against the biothreat pathogen *Yersinia pestis*, both *in vitro* (MIC range < 0.03–2 μg mL^−1^) and in mice (90% survival at 30 days post-challenge, dosing 200 mg kg^−1^ GT-1 and 300 mg kg^−1^ GT-055) (Zumbrun *et al*. 2018). GT-055 has also been shown to potentiate GT-1 in strains of *E. coli* with ESBLs that afford greater protection against the latter, such as CTX-M-15, and in strains of *K. pneumoniae* with DHA-1 AmpC (Phuong *et al*. 2018).

Boronic acid transition state inhibitors (BATSIs) are a novel class of BLIs with activity against serine β-lactamases. BATSIs are characterised by the presence of a boronic acid functionality, cyclic or acyclic, within the molecule; the electrophilic nature of the boron atom imitates the electrophilic carbonyl centre of a β-lactam ring, but nucleophilic attack by the catalytic serine residue of a β-lactamase generates a tetrahedral enzyme-BATSI adduct, inhibiting the enzyme in a competitive, reversible manner (Rojas *et al*. 2016).

**Figure 4.**
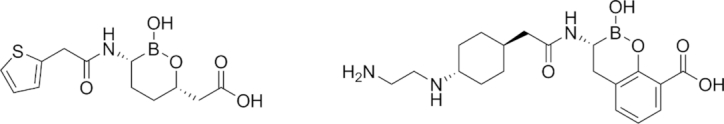
The BATSIs. Structure of vaborbactam (left) and VNRX-5133 (right) (Hecker *et al*. 2015; Docquier *et al*. 2018).

Of the BATSIs, vaborbactam (RPX7009; developed by Rempex Pharmaceuticals, Inc., A Subsidiary of The Medicines Company; Fig. 4) is currently the furthest advanced with respect to clinical development. Hecker *et al*. report that it shows inhibition of a broad spectrum of class A, C and D enzymes, including KPC, CTX-M, SHV, and CMY, and improves upon both clavulanic acid and tazobactam against KPC-2, P99 and CMY-2 (Hecker *et al*. 2015). Originally partnered with the carbapenem biapenem (RPX2003, also developed by Rempex Pharmaceuticals) (Livermore and Mushtaq 2013), Goldstein and co-workers found the combination improved upon biapenem monotherapy against select anaerobic Gram-negative bacteria (*Bacteroides fragilis*, *Bacteroides ovatus* and *Fusobacterium mortiferum*) but not significantly for any anaerobic Gram-positive bacteria tested (Goldstein *et al*. 2013). Livermore and Mushtaq found vaborbactam itself to lack any direct antibacterial activity, but found the biapenem-vaborbactam combination to improve upon biapenem alone against a panel of 145 KPC-positive Enterobacteriaceae isolates (94.4% of isolate MICs below 1 μg mL^−1^ for combination vs. 5.5% for biapenem alone) at a vaborbactam concentration of 8 μg mL^−1^ (Livermore and Mushtaq 2013). A combination with the cephalosporin cefepime at 4 μg mL^−1^ vaborbactam was active *in vitro* against a panel of 13 Enterobacteriaceae expressing class A, C and D β-lactamases, showing 2–256-fold potentiation of cefepime across the panel, and vaborbactam also potentiated the carbapenems biapenem, meropenem, ertapenem and imipenem up to 512-fold versus a panel of 11 Enterobacteriaceae expressing class A carbapenemases (Hecker *et al*. 2015).

Lapuebla and co-workers evaluated the meropenem-vaborbactam combination (Carbavance®) at 8 μg mL^−1^ vaborbactam *in vitro* against a collection of 4500 Gram-negative clinical isolates from 11 New York City hospitals and found it to be highly active against KPC-producing Enterobacteriaceae including *E. coli*, *Enterobacter* spp. and *K. pneumoniae*. 98.5% of KPC-producing Enterobacteriaceae strains showed MICs below 1 μg mL^−1^, but vaborbactam did not potentiate meropenem against *A. baumannii* and *P. aeruginosa*. In addition, *K. pneumoniae* isolates with reduced expression of genes *ompK35* and *ompK36* were less susceptible to the combination (Lapuebla *et al*. 2015). A number of subsequent studies (Castanheira *et al*. 2016c; Castanheira *et al*. 2017b; Lomovskaya *et al*. 2017; Hackel *et al*. 2018; Pfaller *et al*. 2018) were in agreement with these findings; together, they note a number of additional factors that contribute to increased MICs for meropenem-vaborbactam in *K. pneumoniae* isolates, including possession of MBLs, reduced expression of ompK37, increased expression of the AcrAB-TolC efflux system (Castanheira *et al*. 2016c) and possession of class B or D carbapenemases (Lomovskaya *et al*. 2017). Sun and co-workers found the frequency of spontaneous resistance to the combination to be < 1 × 10^−8^ in 77.8% of a panel of KPC-producing *K. pneumoniae* strains at 8 μg mL^−1^ of both meropenem and vaborbactam (Sun, Deng and Yan 2017). Reports by Weiss *et al*. and Sabet *et al*. demonstrated that the combination is active in a murine pyelonephritis model (Weiss *et al*. 2018a), an *in vitro* hollow fibre model (Sabet *et al*. 2018a) and murine thigh and lung infection models (Sabet *et al*. 2018b), respectively.

Vaborbactam has completed a number of phase I clinical trials alone (Griffith *et al*. 2016) and in combination with biapenem (ClinicalTrials.gov, NCT01772836) and meropenem (ClinicalTrials.gov, NCT02073812) (Rubino *et al*. 2018a; Rubino *et al*. 2018b). The meropenem-vaborbactam combination has completed two phase III trials, evaluating its use against complicated urinary tract infections (Kaye *et al*. 2018) and infections of carbapenem-resistant Enterobacteriaceae (Wunderink *et al*. 2018), and the FDA approved the combination for the former indication in August 2017 (McCarthy and Walsh 2017). Meropenem-vaborbactam is currently being evaluated for treatment of hospital-acquired and ventilator-associated bacterial pneumonia in a TANGO III trial (ClinicalTrials.gov, NCT03006679).

Other notable work in the field of BATSIs includes that of VenatoRx Pharmaceuticals, who are developing the BATSI VNRX-5133 (Fig. 4) (Docquier *et al*. 2018). Patented in 2016 (Burns *et al*. 2016), VNRX-5133 inhibits class A, C and D serine β-lactamases and VIM/NDM class B MBLs in both carbapenem-resistant Enterobacteriaceae and *P. aeruginosa*. X-ray crystallography conducted on VNRX-5133 bound to the class A serine ESBL CTX-M-15 found the compound to bind the enzyme at the catalytic serine residue *via* the boron atom, which was sp^3^ hybridised. Similar experiments with VIM-2 found that the boron adopted the same hybridisation state, with its hydroxyl group interacting with Zn1 and the Asn233 residue and the cyclic oxygen atom interacting with Zn2 (Docquier *et al*. 2018). Steady state inhibition studies confirmed VNRX-5133 to be a potent competitive VIM-2 inhibitor (Daigle *et al*. 2018). A combination with cefepime appears promising; of two panels, one of 1120 recent Enterobacteriaceae isolates and another of 155 NDM- or OXA-positive Enterobacteriaceae isolates, 99% and 81% were inhibited at or below the cefepime breakpoint, respectively (Hackel and Sahm 2018, Kazmierczak *et al*. 2018). Cefepime activity was also restored to below breakpoint (8 μg mL^−1^) in 93.1% of 245 clinical CRE isolates (Tyrrell *et al*. 2018), 70% of 817 isolates of *P. aeruginosa* resistant to cefepime, meropenem or both (Estabrook *et al*. 2018) and 90% of 29 ESBL- and carbapenemase-producing *P. aeruginosa* isolates (Donnelly *et al*. 2018). 98.3% of 1066 Enterobacteriaceae urinary tract infection isolates resistant to co-amoxiclav and levofloxacin were susceptible to the combination (Hackel and Sahm 2018). Potentiation of cefepime between 8 and over 2048-fold by VNRX-5133 against individual Enterobacteriaceae strains possessing CTX-M-15, KPC, VIM-1, NDM-1 and OXA-48 enzymes has also been reported (Hamrick *et al*. 2018). The combination has proved effective in murine bacteremia (Weiss *et al*. 2018b), lung (Weiss *et al*. 2018c), urinary tract (Weiss *et al*. 2018d) and neutropenic thigh infection models (Georgiou *et al*. 2018), has completed a phase I study in healthy volunteers (ClinicalTrials.gov, NCT02955459) and is currently undergoing a phase I drug-drug interaction study (ClinicalTrials.gov, NCT03332732).

Very few effective inhibitors of MBLs have been discovered to date, with none in clinical use currently. Unlike the serine β-lactamases, MBLs achieve hydrolysis of β-lactam antibiotics *via* a divalent metal cofactor, often zinc (Davies and Abraham 1974; Wommer *et al*. 2002). These enzymes are of special concern because of their activity against penicillins, cephalosporins and carbapenems (covering both widely used and last-resort antibiotics) and their resistance to the actions of all BLIs in current clinical use (Palzkill 2013).

A potential clinically useful MBL inhibitor is aspergillomarasmine A, a natural fungal product that has shown efficacy against the MBLs NDM-1 and VIM-2 and has been shown to resensitise MBL-positive strains of *Pseudomonas* spp., *Acinetobacter* spp. and Enterobacteriaceae to meropenem. Aspergillomarasmine A restored meropenem activity in CD1 mice infected with NDM-1-producing *K. pneumoniae* (>95% survival rate after 5 days, single dose of aspergillomarasmine A and meropenem combination). It is thought to act as a Zn^2+^ ion chelator, achieving demetallation and thus inactivation of MBLs (King *et al*. 2014).

Discovered in Japan by Meiji Seika Kaisha Ltd., ME1071 is a maleic acid derivative (Yamada *et al*. 2013) and selective MBL inhibitor capable of potentiating carbapenems and ceftazidime against MBL-positive *P. aeruginosa* (Ishii *et al*. 2010). Work by Livermore *et al*. demonstrates that, regardless of partner carbapenem, ME1071 achieved its greatest levels of potentiation against strains possessing IMP-type MBLs and its lowest levels against NDM-type MBLs (Livermore *et al*. 2013). Combined with biapenem, ME1071 significantly prolonged the survival of mice infected with MBL-positive *P. aeruginosa* compared with both control and biapenem monotherapy groups (*P* < 0.05) (Yamada *et al*. 2013).

The metal chelating agents 1,4,7-triazacyclononane-1,4,7-triacetic acid and 1,4,7,10-tetraazacyclod-odecane-1,4,7,10-tetraacetic acid have been reported by Somboro *et al*. to inhibit NDM, VIM and IMP-type MBLs (Somboro *et al*. 2015; Zhang *et al*. 2018).

Wang *et al*. have reported that a number of Bi(III)-containing compounds, including colloidal bismuth subcitrate (CBS), inhibit a large variety of B1 MBLs (at 32 μg mL^−1^, CBS potentiated meropenem 16-fold against NDM-1-positive *Citrobacter freundii*, 64-fold against VIM-2-positive *E. coli* BL21 and 8-fold against IMP-4-positive *E. coli* BL21). IC_50_ values against NDM-1, VIM-2 and IMP-4 for CBS were 2.81  ±  0.34 μM, 3.55  ±  0.78 μM and 0.70  ±  0.08 μM, respectively. The study concluded that such compounds achieve MBL (NDM-1) inhibition through binding to a cysteine residue (shown to be Cys208 in NDM-1 *via* a C208A mutant), leading to release of Zn(II) from the enzyme. CBS was also observed to suppress resistance mutations in NDM-1-positive bacteria; when added to meropenem (½ MIC concentration) against the NDM-1-positive *E. coli* strains NDM-HK, CBS reduced the mutation frequency from approximately 4  ×  10^−7^ (no CBS) to 1  ×  10^−10^ (256 μg mL^−1^ CBS) (Wang *et al*. 2018).

Recent studies by Spyrakis *et al*. and Cain *et al*. have demonstrated *in silico* screening as a possible method of MBL inhibitory discovery and development. Spyrakis and co-workers docked a commercially available library of compounds with available crystal structures for NDM-1 (protein data bank ID 3Q6X and 3SPU) to identify a number of non-β-lactam compounds with MBL-inhibitory activity (compound 1 *K*_i_ 0.72 ±  0.014 μM) (Spyrakis *et al*. 2018). In contrast, Cain *et al*. used the *de novo* molecular design program SPROUT to generate possible ligands for a crystal structure of NDM-1 (protein data bank ID 3Q6X) and found 2-(mercaptomethyl)benzoic acid as a putative NDM-1 substrate-competitive inhibitor. Further modification of this scaffold resulted in a number of analogues with potent B1 MBL inhibitory activities (compound 5; NDM-1 IC_50_ 0.31 ±  0.05 μM, VIM-2 IC_50_ 0.07 ±  0.05 μM, IMP-1 IC_50_ 0.14 ±  0.05 μM) (Cain *et al*. 2018).

Two other novel classes of BLI being developed are O-acyl and O-phosphyl hydroxamates. Tilvawala and Pratt assessed the effectiveness of N-phenylcarbonyl and N-tertbutoxycarbonyl derivatives of the cyclic O-acyl-hydroxamic acid, 3H-benzo[d][1,2]oxazine-1,4-dione. These compounds are prodrugs with no BLI activity which spontaneously hydrolyse in aqueous solution to produce O-phthaloyl hydroxamic acids with serine-BLI activity, and both can subsequently and reversibly cyclise in solution to form phthalic anhydride, another BLI. For both derivatives, both the O-phthaloyl hydroxamic acid and phthalic anhydride forms can react to form covalent phthaloyl-enzyme complexes and it was found that incubation of either compound with P99 β-lactamase resulted in inhibition of the enzyme (t_1/2_ for turnover of CENTA™ (50 µM) by enzyme (1.0 nM) alone (100 s), in presence of phthalic anhydride (500 s at 30 mM) and in presence of O-phthaloyl hydroxamic acids (>1500 s for both at 10 mM phthalic anhydride, 10 mM hydroxamic acid)). Inhibition was observed to be transient; this was ascribed to hydrolysis of the phthaloyl-enzyme complexes leading to reactivation of the β-lactamase (Tilvawala and Pratt 2013).

Cyclobutanone derivatives of β-lactams have received attention as potential serine- and MBL inhibitory compounds (Johnson *et al*. 2008; Devi and Rutledge 2017). Johnson and co-workers tested a number of such compounds against representative β-lactamases from classes A-D (KPC-2, IMP-1, GC1 and OXA-10, respectively); micromolar inhibitory activity was observed against KPC-2 and GC1, with activity against IMP-1 and OXA-10 less pronounced (Johnson *et al*. 2010).

#### Aminoglycoside-modifying enzyme inhibitors

The aminoglycosides are a family of bacterial protein synthesis inhibitors that bind to the A site of the prokaryotic 70S ribosome and possess bactericidal activity (Doi and Arakawa 2007). Aminoglycoside resistance is a major concern because of the several important uses of aminoglycoside antibiotics, including treatment of infections of *Mycobacterium tuberculosis*. While the initial treatment for *M. tuberculosis* infections usually consists of a combination regimen including rifampicin, ethambutol, pyrazinamide and isoniazid, streptomycin is a suitable alternative when isoniazid resistance has been established or if the patient has any tolerability issues with the initial regimen. Amikacin is a suitable second-line option when there are further issues with resistance or side effects caused by the first-line drugs (Rojano, Caminero and Hayek 2019).

Resistance to aminoglycosides may arise through several different mechanisms, including extrusion by efflux pumps, reduced outer membrane (OM) permeability, target modification and enzymatic inactivation. Target modification may occur by methylation of specific nucleotides within the 16S rRNA. This resistance mechanism was initially identified in aminoglycoside-producing species such as *Streptomyces* spp., providing them with intrinsic resistance against the antibiotics they produce. This mechanism was subsequently identified in several strains of clinically relevant bacteria such as *P. aeruginosa* (Doi and Arakawa 2007). However, the most prevalent aminoglycoside resistance mechanism is enzymatic inactivation. As with the β-lactamases, these modifying enzymes may be further subdivided into several groups, members of which achieve the same effects through slightly different mechanisms. The three groups of aminoglycoside modifying enzymes are the aminoglycoside acetyltransferases (AACs), aminoglycoside nucleotidyltransferases nd aminoglycoside phosphotransferases (APHs). AACs function by catalysing the acetylation of primary amine groups within the aminoglycoside molecules, using acetyl coenzyme A (CoA) as a donor substrate. aminoglycoside nucleotidyltransferases are responsible for mediating the transfer of an adenosine monophosphate group to a hydroxyl group in the aminoglycoside molecule, using ATP as a donor substrate, while APHs catalyse the transfer of a phosphate group to the aminoglycoside molecule (Ramirez and Tolmasky 2010). Several promising inhibitors of these enzymes have been developed, but none have yet entered clinical use.

In 1997, Hon and co-workers reported that APH (3’) enzymes (EC 2.3.1.81) show unusually high structural similarity to eukaryotic protein kinases despite minimal sequence homology (Hon *et al*. 1997). This inspired the testing of protein kinase inhibitors as inhibitors of APHs. Selective inhibition is an important requirement for any such repurposed compound, since the inhibitor must be able to distinguish between eukaryotic protein kinases and APHs. Stogios *et al*. identified pyrazolopyrimidine compounds with selective inhibitory activity against the enzyme APH (3’)-Ia, which plays a large role in Gram-negative resistance against aminoglycoside antibiotics, and suggested that these can be further developed to provide a new option for combatting aminoglycoside resistance (Stogios *et al*. 2013).

Another strategy is the use of bisubstrate analogues, consisting of the aminoglycoside antibiotic and CoA. This strategy was successfully carried out with gentamicin; the bisubstrate showed inhibitory activity *in vitro* (but not *in vivo*) towards the enzyme AAC(3)-I. It was suggested that this was due to poor compound influx into Gram-negative bacterial cells (Williams and Northrop 1979). Since this discovery, several aminoglycoside-CoA conjugates have been synthesised, with varying functional groups. It was found that amide-linked bisubstrates that contained sulfoxide and sulfone functionalities showed effective inhibition of AAC(6)-Ii (EC 2.3.1.82) at nanomolar concentrations (Gao *et al*. 2008).

Boehr *et al*. screened several antimicrobial peptides against the aminoglycoside-modifying enzymes APH(3’)-IIIa (EC 2.7.1.95), AAC(6’)-Ii and AAC(6’)-APH(2″) (EC 2.3.1.81). The bovine peptide indolicidin (primary sequence H-ILPWKWPWWPWRR-NH_2_) and its analogues CP11CN (H-ILKKWPWWPWRRK-NH_2_) and CP10A (H- ILAWKWAWWAWRR-NH_2_) were all demonstrated to inhibit AAC(6’)-Ii, with IC_50_ values of 13, 23 and 4.4 μM respectively, and APH(3’)-IIIa, with IC_50_ values of 11, 51 and 11 μM respectively (Boehr *et al*. 2003).

### Membrane permeabilisers

Gram-negative bacteria are intrinsically resistant to several antibiotic classes because of the presence of a second, OM compared to Gram-positive bacteria which these antibiotics cannot penetrate. The Gram-negative bacterial envelope consists of three components; an inner membrane which surrounds the organelles, an OM and a periplasmic region between the two membranes containing a peptidoglycan layer (Silhavy, Kahne and Walker 2010). The OM consists mainly of lipopolysaccharides (LPS), which are made up of three parts; a polysaccharide referred to as the O-antigen, a core domain consisting of an oligosaccharide component and a lipid region referred to as lipid A (Raetz and Whitfield 2002). This LPS layer is stabilised by cross-linking, enabled by divalent cations such as Mg^2+^ and Ca^2+^ (Zabawa *et al*. 2016). The OM contains porins, water-filled protein channels that facilitate entry of hydrophilic molecules into the bacterial cell; mutations in Gram-negative bacteria resulting in reduced porin expression can reduce influx of hydrophilic drugs into these bacteria. This method of antibacterial resistance has been confirmed in several clinically relevant bacterial species, such as *P. aeruginosa* (Fernandez and Hancock 2012).

Besides directly damaging the cell membrane, various other methods have been suggested to increase rates of antibiotic influx in bacterial cells, such as the use of liposomal drug preparations (Torres *et al*. 2012). However, it is the use of membrane permeabilisers, compounds that make the Gram-negative OM more permeable to facilitate increased antibiotic influx, that will be reviewed herein. Membrane permeabilisers can function by chelating and removing divalent cations from the OM and/or (in the case of permeabilisers with a net cationic charge) associating with the negatively charged OM to disrupt it, causing a breakdown of OM structure (Zabawa *et al*. 2016). The effectiveness of putative membrane permeabilisers can be assessed by measuring the level of uptake of substances that would not normally be able to penetrate the Gram-negative OM, such as a hydrophobic probe. The fluorescent dye N-phenyl-1-napthylamine (NPN) is used for this purpose; an increase in fluorescence indicates increased incorporation of NPN into the OM of the pathogen and thus increased OM permeability (Lee *et al*. 2004). Besides enabling increased influx of antibiotics, membrane permeabilisation alone can be sufficient to cause bacterial lysis; as such, several of the compounds mentioned in this section also have direct antibacterial activity (Zabawa *et al*. 2016).

#### The polymyxins

**Figure 5.**
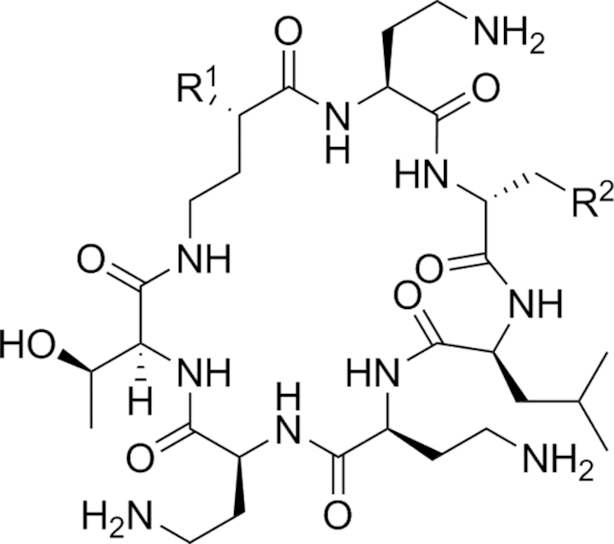
The Polymyxins. General structure of the polymyxins (Velkov *et al*. 2010).

Polymyxins (see Fig. 5 and Table 2), including polymyxin B and polymyxin E (colistin), are antibiotics that function through disruption of the Gram-negative OM. First reported in 1947 (Ainsworth, Brown and Brownlee 1947; Benedict and Langlykke 1947; Stansly, Shepherd and White 1947), the polymyxins are pentacationic lipopeptides consisting of a cyclic peptide attached to a long fatty acid chain. Colistin itself was available for treatment of Gram-negative bacterial infections from 1959 (Ross, Puig and Zaremba 1959), though clinical use decreased from the 1970s to the 1990s because of reports of neurotoxicity and nephrotoxicity (Vaara 1992; Li *et al*. 2005; Falagas and Kasiakou 2006). While Falagas and Kasiakou report that adverse events related to current polymyxin use are less frequent than reported in older literature, possibly due to better understanding of appropriate dosing regimen and the avoidance of simultaneous administration of nephrotoxic and/or neurotoxic drugs (Falagas and Kasiakou 2006), increased colistin use in recent years has been primarily driven by the onset of resistance to β-lactams, aminoglycosides and quinolones in Gram-negatives (Livermore 2002). Polymyxins interact electrostatically with the OM to displace Mg^2+^ and Ca^2+^ cations from their binding sites to disrupt membrane integrity, causing cell damage and also facilitating the influx of other molecules, including other antibiotics (Landman *et al*. 2008). Lin *et al*. found that azithromycin, ineffective against Gram-negative rods, showed synergy with colistin; the combination was effective against MDR-isolates of *P. aeruginosa, K. pneumoniae* and *A. baumannii* (Lin *et al*. 2015). Synergistic combinations of colistin with other drugs have also been reported; Lee and co-workers found that the combination of colistin and rifampicin at clinically-relevant concentrations was additive or synergistic against MDR strains of *A. baumannii* and suppressed the emergence of colistin resistance (Lee *et al*. 2013).

**Figure 6.**
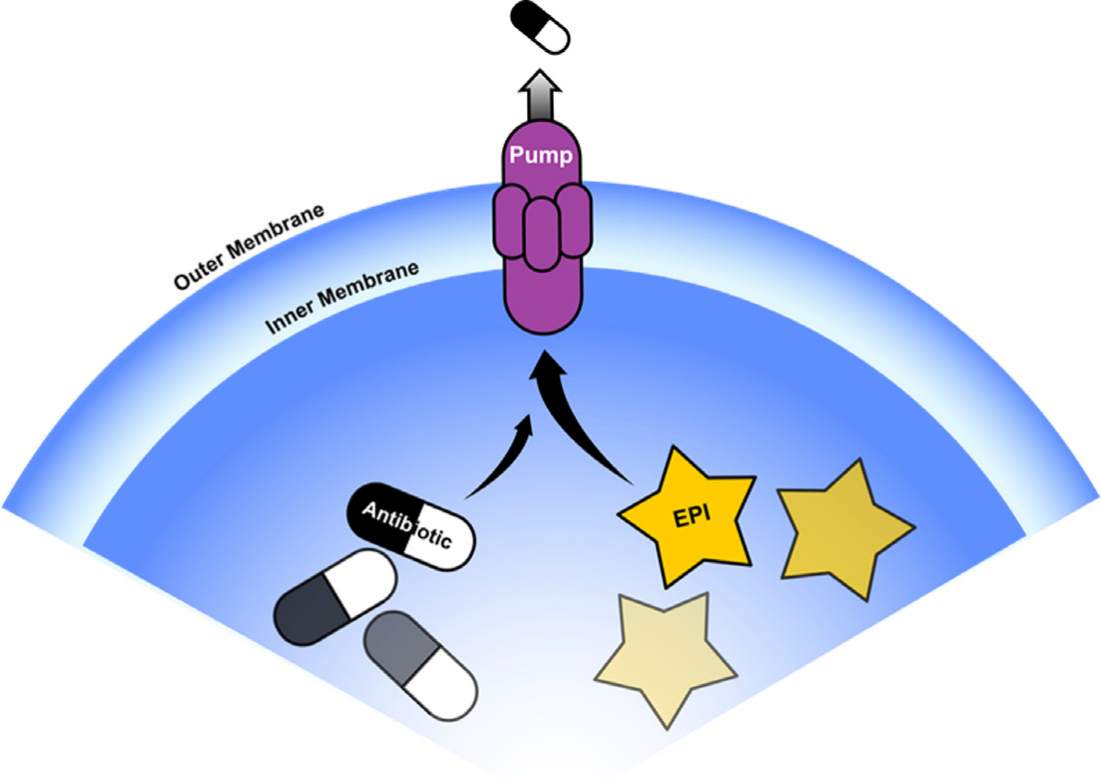
Efflux Substrate Competition. Competition for pump binding between discrete EPI and antibiotic molecules.

**Table 2.** Structures of the polymyxins and their derivatives (Vaara 1988; Vaara *et al*. 2010; Velkov *et al*. 2010).

Name	R^1^ group	R^2^ group
Polymyxin B1		Ph
	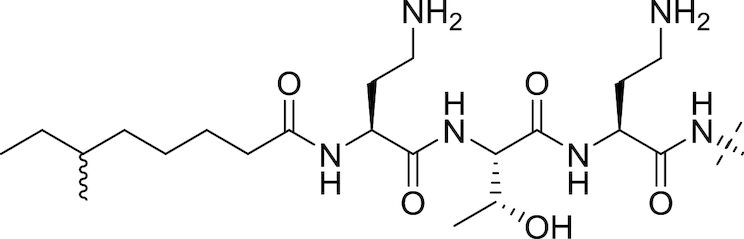	
Polymyxin B2	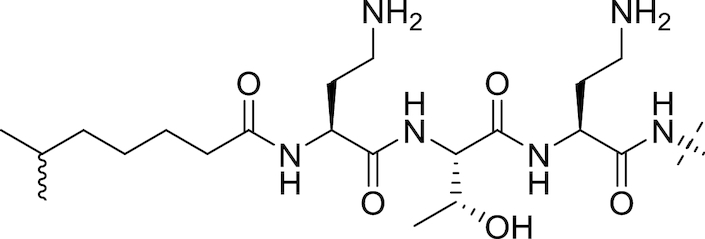	Ph
Colistin A	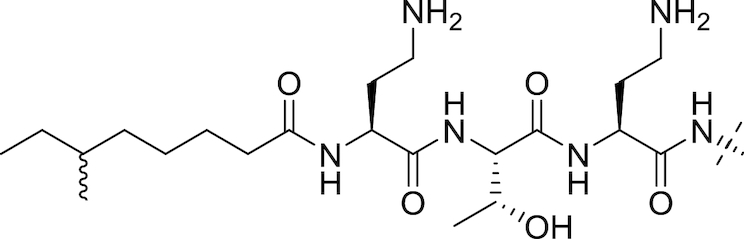	^i^Pr
Colistin B	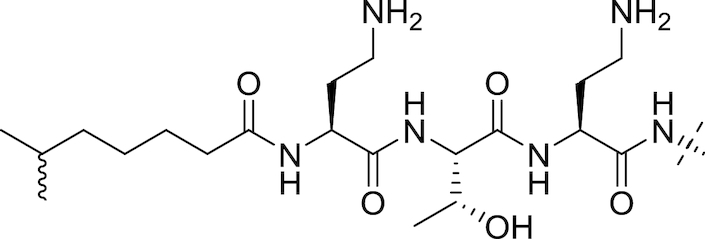	^i^Pr
PMBN	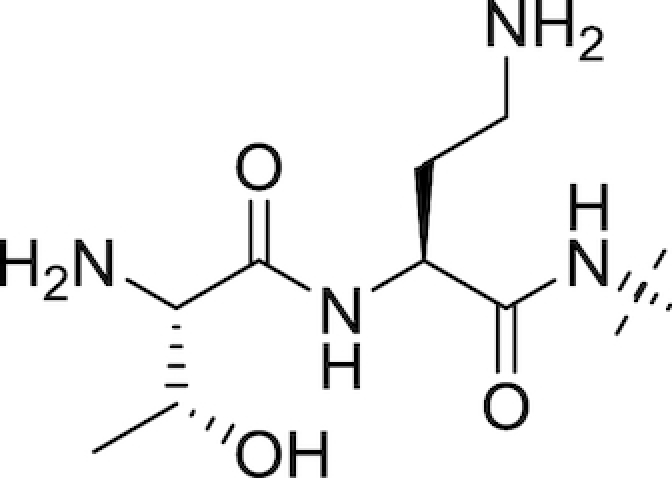	Ph
SPR741	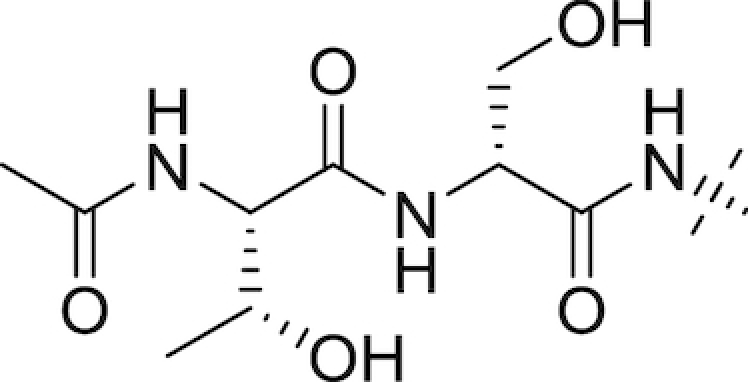	Ph

The membrane permeabilising effects of colistin towards pandrug-resistant Gram-negative bacteria have been investigated. Pandrug-resistant bacteria refers to bacteria that are resistant to all anti-pseudomonal drugs (penicillins, cephalosporins, carbapenems, monobactams, quinolones and aminoglycosides) except the polymyxins, although some commentators include the polymyxins in this definition. Mohamed *et al*. measured the effect of 50 μg mL^−1^ of colistin on the cytoplasmic membranes of four pandrug-resistant clinical isolates (*A. baumannii* A182, *P. aeruginosa* P103, *K. pneumoniae* K103 and *E. coli* E9). This concentration of colistin was far in excess of the MICs for the isolates (range 0.625–1.25 μg mL^−1^) and resulted in net membrane leakage in all four isolates. The haemolytic effect of colistin on human red blood cells was also assessed; at a concentration of 12.5 μg mL^−1^, colistin caused approximately 1.3% haemolysis, confirming its selectivity towards bacterial membranes (Mohamed *et al*. 2016).

#### Polymyxin derivatives

Increasing levels of polymyxin resistance globally (Cannatelli *et al*. 2016; Lee *et al*. 2016; Liu *et al*. 2016; Rapoport *et al*. 2016; Skov and Monnet 2016; Kluytmans 2017; Haeili, Kafshdouz and Feizabadi 2018) necessitate the development of novel alternatives. Efforts to develop polymyxin derivatives as ARBs to potentiate the actions of antibiotics have been spearheaded in recent decades by Prof. Martti Vaara and co-workers. Chihara *et al*. first described in 1972 the production of a novel, truncated form of polymyxin B, later termed polymyxin B nonapeptide (PMBN; Table 2) (Vaara 1988). PMBN lacks the fatty acid and terminal diaminobutyric acid moieties of polymyxin B required for bactericidal activity (Vaara 1988), but it does retain the OM permeabilising character of the latter, a fact first demonstrated by Vaara in 1983 (Vaara and Vaara 1983). Together with subsequent work, they showed it capable of enhancing penetration of hydrophobic antibiotics, including erythromycin, clindamycin, rifampicin, fusidic acid, novobiocin and cloxacillin, in most polymyxin-susceptible Gram-negative bacteria, including MDR *E. coli*, *K. pneumoniae* and *P. aeruginosa* (Viljanen and Vaara 1984). Ofek *et al*. tested several polymyxin-susceptible strains of *E. coli*, *K. pneumoniae*, *P. aeruginosa* and *Salmonella typhimurium* with PMBN and found that it sensitised the majority of them to six different antibiotics (ampicillin, erythromycin, lincomycin, nafcillin, novobiocin and vancomycin). The exceptions were a single strain of *P. aeruginosa* resistant to nafcillin, one *E. coli* strain and two *K. pneumoniae* strains resistant to vancomycin; it was suggested that these strains possess resistance mechanisms unrelated to permeability (Ofek *et al*. 1994). Zabawa *et al*. posit that development of PMBN ultimately stalled because of similar levels of nephrotoxicity to polymyxin B in rats (Zabawa *et al*. 2016).

Many membrane permeabilising agents require a net positive charge to exert their effects. However, in a second generation of polymyxin B derivatives, Vaara *et al*. showed that analogues with a reduced number of positive charges display a concomitant reduction in nephrotoxicity. The derivatives, which contain only three positive charges at physiological conditions instead of the usual five, had a lower affinity for rat kidney border brush membranes than polymyxin B. Antibacterial activity was not necessarily compromised; MICs of one derivative, NAB739, were comparable to those of polymyxin B against 17 different *E. coli* strains (range 0.5–1 μg mL^−1^ for NAB739, range 0.25–1 μg mL^−1^ for polymyxin B) and were 2–8 fold higher than the corresponding polymyxin B MICs for strains of *Klebsiella oxytoca*, *K. pneumoniae*, *E. cloacae*, *C. freundii*, *A. baumannii* and *P. aeruginosa*. Furthermore, at sub-MIC concentrations, NAB739 potentiated the activity of several antibiotic drugs against *A. baumannii*, including rifampicin, vancomycin and clarithromycin (Vaara *et al*. 2008).

Another second generation derivative, NAB741 (Table 2), showed strong synergism with rifampin and clarithromycin against resistant strains of *E. coli*, *K. pneumoniae*, *E. cloacae* and *A. baumannii* and sensitised *E. coli* and *E. cloacae* strains to azithromycin, mupirocin, fusidic acid and vancomycin (Vaara *et al*. 2010). Further studies detailed reduced cytotoxicity in NAB741 versus polymyxin B, with the former found to have 32-fold cytotoxicity towards LLC-PK1 cells than the latter (Mingeot-Leclercq *et al*. 2012). These second generation derivatives were patented by Vaara, Vaara and Northern Antibiotic Ltd (Vaara and Vaara 2009) in 2009 and subsequently licensed to Spero Therapeutics, where NAB741 was given the code SPR741. Corbett *et al*. reported that 8 μg mL^−1^ of SPR741 was sufficient to potentiate thirteen antibiotics (azithromycin, clarithromycin, dalfopristin, erythromycin, fidaxomicin, fosfomycin, fusidic acid, mupirocin, novobiocin, ramoplanin, retapamulin, rifampicin and telithromycin) between 32 and 8192-fold against *E. coli* ATCC 25 922 (Corbett *et al*. 2017). At 16 μg mL^−1^, SPR741 potentiated 10 antibiotics (azithromycin, clarithromycin, erythromycin, fusidic acid, mupirocin, novobiocin, retapamulin, rifampicin, telithromycin and vancomycin) between 32 and 128-fold against *K. pneumoniae* ATCC 43 816, and 8 antibiotics (clarithromycin, dalfopristin, erythromycin, fusidic acid, ramoplanin, retapamulin, rifampicin and teicoplanin) between 32 and 128-fold against *A. baumannii* NCTC 12 156 (Corbett *et al*. 2017). Further work by Zurawski and co-workers using a panel of 28 extensively drug-resistant strains of *A. baumannii* found that a combination of 1 μg mL^−1^ rifampicin combined with 4 μg mL^−1^ SPR741 was sufficient to inhibit growth in 27 of the strains (96%). The exception, strain AB3927, was significantly more resistant to rifampicin (MIC > 256 μg mL^−1^) compared to the other strains (MIC range 2–16 μg mL^−1^) (Zurawski *et al*. 2017). As of December 2017, SPR741 has completed two phase I clinical trials; a 64-person, first-in-man study to assess safety and tolerability (ClinicalTrials.gov, NCT03022175) and a 27-person trial evaluating separate combinations of SPR741 and ceftazidime, piperacillin/tazobactam and aztreonam (ClinicalTrials.gov, NCT03376529).

#### Other permeabilisers

Antimicrobial peptides, an umbrella term encompassing a diverse array of compounds produced by a variety of organisms to combat infections of pathogenic microorganisms, have been investigated for use as ARBs. The temporins, the first 10 members of which were isolated from the skins secretions of the European common frog *Rana temporaria*, are a notable example; Giacometti *et al*. investigated the activity of temporin A against *Enterococcus faecalis* and reported both direct activity and synergism with imipenem and co-amoxiclav (Simmaco *et al*. 1996; Giacometti *et al*. 2005). LL-37, a cathelicidin class antimicrobial peptide found in humans, was found by Lin and colleagues to potentiate the macrolide antibiotic azithromycin and the combination to be synergistic at sub-MIC concentrations against MDR strains of *P. aeruginosa*, *K. pneumoniae* and *A. baumannii* (Lin *et al*. 2015). Synthetic Antimicrobial peptides appear equally attractive ARB candidates; Lainson *et al*. have described synergy between a novel bivalent peptide, ASU014, and the narrow spectrum β-lactam oxacillin against MRSA at sub-MIC concentrations of both compounds both *in vitro* and in a skin infection model (Lainson *et al*. 2017). A more thorough discussion of antimicrobial peptides in this capacity can be found in previous summaries (Kosikowska and Lesner 2016).

Peptidomimetics are synthetic compounds that mimic the membrane permeabilization mechanism of action of antimicrobial peptides, but are stable to enzymatic degradation. A peptidomimetic library was designed by Radzishevsky *et al*. with alternating acyl chains and cationic amino acids, called oligo-acyl-lysyls, with the purpose of avoiding the formation of undesirable secondary structures. Compound C_12_K-7α_8_ exhibited significant antimicrobial activity against several Gram-negative bacteria, including *Klebsiella* spp. and *Pseudomonas* spp., and showed no increase in MICs after several subcultures, indicating a low probability of resistance emerging. Furthermore, C_12_K-7α_8_ showed minimal haemolytic activity up to at least 156 μM, a concentration 100-fold higher than its MIC for several bacteria (Radzishevsky *et al*. 2007). A subsequent study from the same group found that C_12_K-7α_8_ at ½ MIC concentration was capable of potentiating several cytoplasm-targeting antibiotics (erythromycin, clarithromycin and tetracycline) up to 256-fold versus four MDR *E. coli* clinical isolates, but was far less efficient at potentiating periplasm-targeting compounds such as β-lactams. The authors concluded that C_12_K-7α_8_ synergises with efflux-afflicted antibiotics to increase their influx into the bacterial cell, thereby circumventing this resistance mechanism (Livne *et al*. 2010). Further work has established the ability of macromolecular assemblies of C_12_K-7α_8_ to potentiate erythromycin in male ICR mice infected with MDR *E. coli* (Sarig *et al*. 2011). Another oligo-acyl-lysyl, C_12(ω7)_X, has been demonstrated to reduce the MIC of rifampicin against *E. coli*, *K. pneumoniae*, *P. aeruginosa* and *Salmonella enterica* (Jammal *et al*. 2015).

Li and co-workers have demonstrated that cholic acid derivatives are suitable alternatives to polymyxins as ARBs. The derivatives were designed to include structural elements present in the polymyxins, including three primary amine groups and associated hydrophobic chains. Of the derivatives assessed, compound 5 showed particular promise for use as an ARB; while it showed weak direct antibiotic activity against *P. aeruginosa*, *E. coli* and *K. pneumoniae* strains (MIC range 20–50 μg mL^−1^), it showed synergism with erythromycin, novobiocin and rifampicin at concentrations as low as 0.16–5.3 μg mL^−1^ against the aforementioned strains (strains were resistant to all three antibiotics in monotherapy) (Li *et al*. 1999). A further advantage of cholic acid derivatives is their activity against Gram-positive cocci and fungi. Compound 5 had MICs of 3.3, 2.0 and 4.2 μg mL^−1^ against *E. faecalis, S. aureus*, and *Streptococcus pyogenes*, respectively, and had an MIC of 14 μg mL^−1^ against *Candida albicans*, improving on polymyxin B by over four-fold in all cases. However, commercial development of this compound may be complicated because of its significant haemolytic activity (100 μg mL^−1^) (Li *et al*. 1999).

Another example of a permeabiliser is ethylenediaminetetraacetic acid, a chaotropic agent that has been shown to release a large proportion of LPS from the OM. It functions by chelating the divalent cations present in the LPS layer, thereby compromising the integrity and stability of the OM (Vaara 1992). Polyethyleneimine, a cationic polymer, has also shown membrane permeabilising effects. However, instead of releasing LPS, it functions by intercalating into the OM (Helander *et al*. 1997; Helander, Latva-Kala and Lounatmaa 1998). Alakomi *et al*. assessed NPN uptake for several membrane permeabilisers, including ethylenediaminetetraacetic acid and polyethyleneimine, and found that both caused increased NPN uptake in *Pseudomonas* sp. and Stenotrophomonas *nitritireducens* strains. The clinical benefits of polyethyleneimine have been investigated; it was shown in a susceptibility study to induce an increased susceptibility of a *Pseudomonas* sp. strain to erythromycin, novobiocin and fusidin. However, susceptibilities of S. *nitritireducens* and Sinorhizobium *morelense* strains to the same antibiotics did not show similar improvements (Alakomi *et al*. 2006).

Plant-derived phenolic compounds, a group of secondary metabolites abundant in fruit, vegetables and berries, have been shown to possess membrane permeabilising activity. The effect of berry-derived phenolic compounds on the OM permeability of *Salmonella* species was studied by Alakomi *et al*. It was shown that several of these compounds, such as 3-phenylpropionic acid, efficiently permeabilised the membrane as indicated by an increased NPN uptake. Their destabilising effect on the OM, acting on the divalent cations, was confirmed by the addition of MgCl_2_ which partially abolished this effect. The same study also found that the use of organic acids present in berries, such as sorbic and benzoic acid, also resulted in an increased NPN uptake and LPS release (Alakomi *et al*. 2007).

### Efflux pump inhibitors

Bacterial efflux pumps act to decrease intracellular concentrations of antibiotics by pumping antibiotics out of bacterial cells, thereby reducing their effectiveness. The presence of efflux systems has been confirmed in prokaryotic species, archaea and both inferior and superior eukaryotic species (Van Bambeke *et al*. 2003). Their main function is the extrusion of undesirable compounds from cells; these include heavy metals (Nies 2003), organic solvents (Ramos *et al*. 2002), dyes such as ethidium bromide (Kaatz, Seo and Ruble 1993), amphiphilic detergents (Ma *et al*. 1994), biocides (Costa *et al*. 2013), quorum sensing molecules (Pearson, Van Delden and Iglewski 1999) and metabolites (Van Dyk *et al*. 2004) in addition to antibiotics. The presence of efflux pumps and their clinical significance in contributing towards AMR has been confirmed in many bacteria, including *M. tuberculosis* (Ainsa *et al*. 1998) and *P. aeruginosa*. The latter species possesses the tripartite RND systems MexAB-OprM, MexXY-OprM, MexCD-OprJ and MexEF-OprN, which together can extrude fluoroquinolones, tetracycline, chloramphenicol and some β-lactams to achieve a multidrug resistant phenotype (Piddock 2006). Efflux systems have also been implicated in biofilm formation in a number of different bacterial species; a more detailed discussion of this area can be found in previous reviews on the subject (Alav, Sutton and Rahman 2018).

Prokaryotic efflux systems can be categorised into a number of superfamilies according to their energy source, substrates they can act on, composition and membrane-spanning regions. These include the resistance-nodulation-division (RND) family, the major facilitator superfamily (MFS), the ATP-binding cassette (ABC) superfamily, the small multidrug resistance (SMR) family and the multidrug and toxic compound extrusion (MATE) family (Sun *et al*. 2014). Substrate specificity varies between different pumps; some are drug-specific while others act on multiple drugs. Genes for multidrug-extruding pumps are usually found on chromosomes, while drug-specific efflux systems are located on mobile gene elements which can be transferred between different bacteria *via* horizontal gene transfer (Poole 2007).

A popular approach to combatting bacterial efflux systems has been the development of EPI compounds. A diverse array of EPI compounds have been reported to date (Stavri, Piddock and Gibbons 2007; Mahmood *et al*. 2016), both from natural product screening, *de novo* synthetic efforts and in the form of repurposed previously-approved drugs, and these are detailed in Table 3. However, at the time of writing no discrete EPI compound has been approved for clinical use. While unacceptable toxicities would appear to be the most touted explanation, this position may not be unilaterally correct (Lomovskaya 2018; Opperman 2018). Most EPIs investigated thus far adhere to a ‘cork-in-bottle’ method of blocking pumps, serving to physically obstruct passage of substrate molecules through the transporter. But in the absence of compounds able to covalently modify their target pumps, this approach allows for a degree of competition for pump binding between the substrate antibiotic and the inhibitor, with the result that levels of potentiation observed are typically modest (Fig. 6).

**Table 3.** Major classes of EPIs investigated to date.

EPI Class	Biological activity profile	Structure
Natural Sources		
Catechin gallates	Obtained from green tea extracts, catechin gallates have been shown to reverse β-lactam resistance in MRSA, with epicatechin gallate more effective than epigallocatechin gallate. The former managed to reduce the MIC of oxacillin from 64-512 μg mL^-1^ to ≤0.5-1 μg mL^-1^ in three different isolates (Stapleton *et al*. 2004). When incorporated at a concentration of 20 μg mL^-1^, both were able to cause a four-fold decrease in the MIC of norfloxacin in isolates of *S. aureus* and *S. epidermis*. Both compounds were found to possess a weak inhibitory action towards the NorA transporter, with epicatechin gallate being the more potent of the two (Gibbons, Moser and Kaatz 2004). Epigallocatechin gallate also reversed tetracycline resistance in Tet(K)-expressing *S. aureus* and *S. epidermis* strains (Sudano Roccaro *et al*. 2004)	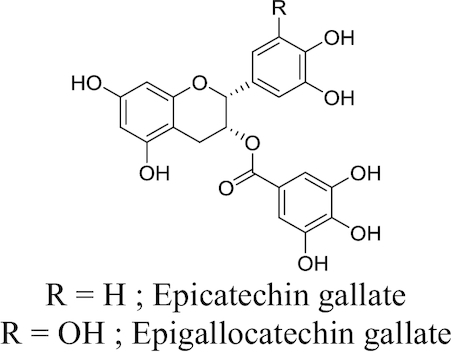
Abietane diterpines	Isolated from the herb *Rosmarinus officinalis*, carnosic acid and carnosol act as potentiators of erythromycin and tetracycline against *S. aureus* strains containing Msr(A) and Tet(k) pumps. At concentrations of 10 µg mL^-1^, both compounds achieved two- and four-fold reductions in the MIC of tetracycline, respectively. Carnosic acid showed synergism with erythromycin, causing an 8-fold reduction in its MIC (Oluwatuyi, Kaatz and Gibbons 2004).	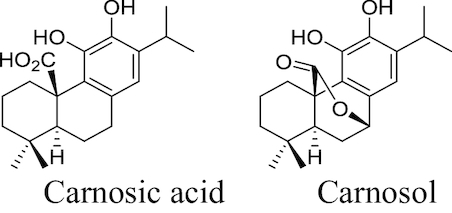
Methoxylated flavones and isoflavones	Baicalein, isolated from the leaves of *Thymus vulgaris*, displays weak antibacterial activity alone (MIC 100 µg mL^-1^) but can reduce the MICs of tetracycline and some β-lactams, including ampicillin and oxacillin, against certain MRSA isolates (Fujita *et al*. 2005). The flavones have shown activity against Gram-positive bacteria, but few reports have been made on their interaction with Gram-negative bacteria (Mahmood *et al*.^2016^).	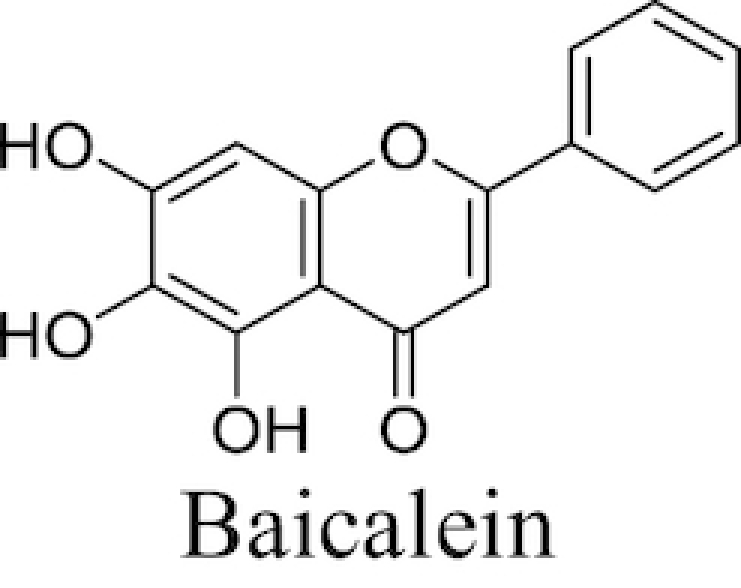
Microbial fermentation products	Compounds EA-371α and EA-371δ were originally isolated from *Streptomyces* fermentation extracts. At 0.625 µg mL^-1^, both compounds caused a four-fold decrease in the MIC of levofloxacin against a strain of *P. aeruginosa* overexpressing the MexAB-OprM efflux system (Lee *et al*. 2001).	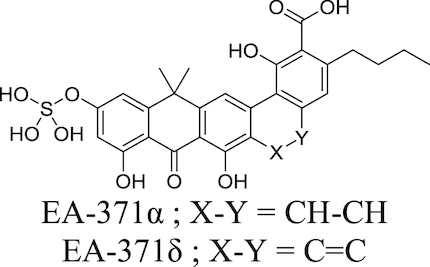
Hetereocyclic macrocycles	Porphyrin pheophorbide A, extracted from *Berberis* spp., can sensitize *S. aureus* to berberine, also extracted from the same plant, and works against the NorA pump. However, several issues including potential toxicity have limited clinical development of this compound and any potential derivatives (Zechini and Versace 2009).	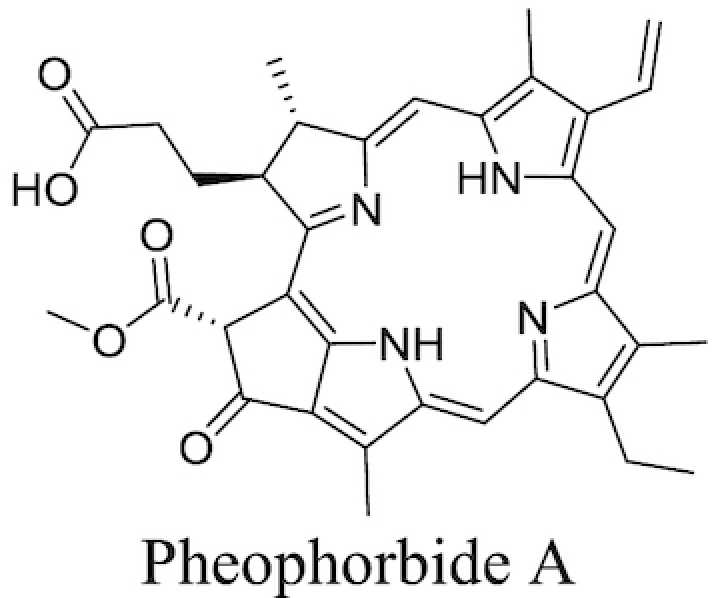
Homoisoflavonoids	Bonducellin, a homoisoflavonoid purified from the roots of *Caesalpinia digyna*, is another compound that has shown potential for use as an EPI. At a concentration of 62.5 µg mL^-1^, bonducellin showed synergistic activity with ethidium bromide against drug-resistant *Mycobacterium smegmatis*, decreasing its MIC eight-fold (Roy *et al*. 2013).	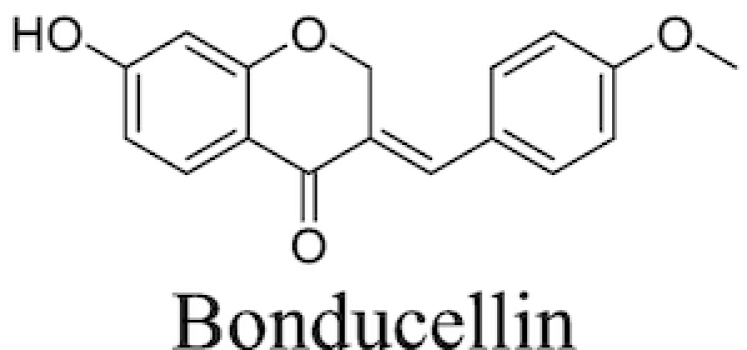
Flavolignans	The flavolignan 5’-methoxyhydnocarpin, extracted from *Berberis* spp., has been identified as an inhibitor of the NorA pump and shows synergism with the fluoroquinolones. Addition of 5’-methoxyhydnocarpin at 10 µg mL^-1^ reduced the MIC of norfloxacin against a wild-type *S. aureus* strain by four-fold, from 1 to 0.25 µg mL^-1^ (Stermitz *et al*. 2000).	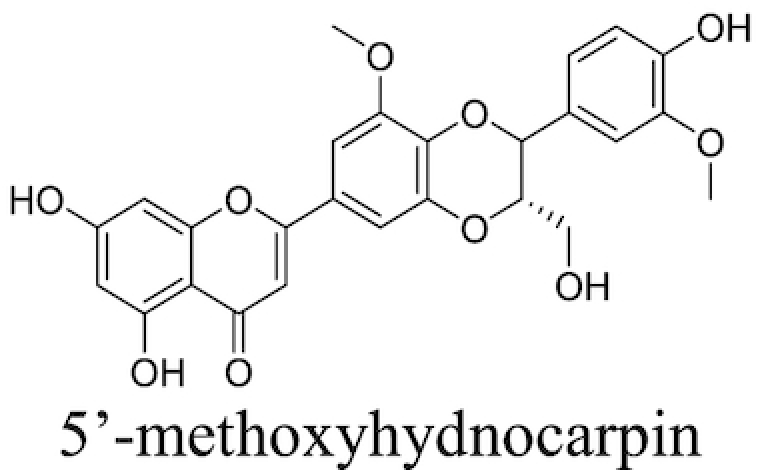
Alkaloids	Reserpine, an indole plant alkaloid extracted from the roots of *Rauvolfia serpentina* and *Rauvolfia vomitoria*, has been shown to be effective in inhibiting the highly homologous (Kaatz *et al*. 1993) NorA and Bmr efflux pumps in *S. aureus* and *Bacillus subtilis*, respectively, although Neyfakh *et al*. reported that two-four-fold greater concentrations of reserpine were required to achieve the same extent of inhibition for the former compared to the latter pump (Neyfakh *et al*. 1993). The primary issue with reserpine, in common with many other EPIs, is its toxicity to mammalian cells. Reserpine has been observed to cause central nervous system disturbances (Pfeifer, Greenblatt and Koch-Wester 1976), limiting its potential for use as an ARB in the clinic. Other alkaloids shown to have EPI character include piperine, obtained from *Piper nigrum* and *Piper longum* (Khan *et al*. 2006), and berberine, found in a variety of plants including *Berberis* spp. (Aghayan, Kalalian Mogadam and Fazli 2017). Piperine has been demonstrated to restore ciprofloxacin susceptibility in certain *S. aureus* strains, causing a four-fold MIC reduction when used at a concentration of 50 µg mL^-1^ (Khan *et al*. 2006). Su and Wang found berberine to potentiate the activity of imipenem *in vitro* against *P. aeruginosa* through inhibiting the tripartite MexXY-OprM efflux pump (Su and Wang 2018).	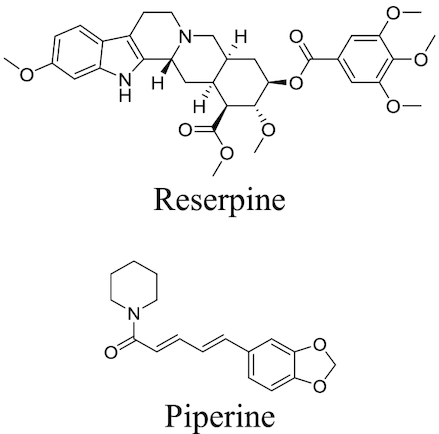
Acyclic sesquiterpene alcohols	Farnesol, an acyclic sesquiterpene alcohol found as a metabolite in both plants and animals, was investigated by Jin and co-workers due to previous reports that it was capable of potentiating antimicrobial agents against strains of both *S. aureus* and *E. coli*. They demonstrated that farnesol is both capable of potentiating the action of ethidium bromide in *Mycobacterium smegmatis* through blocking its efflux and possesses greater intrinsic activity (64 µg mL^-1^) towards *M. smegmatis* than some other EPIs (reserpine 256 µg mL^-1^; verapamil 300 µg mL^-1^) (Jin *et al*. 2010).	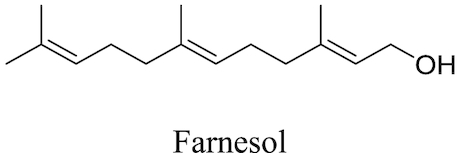
Synthetic Sources		
Peptidomimetics	Arguably the most widely studied, PAβN is a broad-spectrum EPI capable of combatting fluoroquinolone resistance in *P. aeruginosa*. A C-terminal amide dipeptide, Lomovskaya and co-workers showed that at 40 µg mL^-1^ PAβN caused an 8-fold decrease in the MIC of levofloxacin against wild type *P. aeruginosa* strain PAM1020, while a 64-fold reduction was achieved in three strains overexpressing the MexAB-OprM tripartite efflux system (Lomovskaya *et al*. 2001). However, the cytotoxic nature of this compound led Lomovskaya and colleagues at Microcide Pharmaceuticals, Inc. to develop improved analogs between 1995 and 1998, culminating in MC-004124, an EPI with minimised cytotoxicity and acute toxicity and lower serum free drug clearance (Lomovskaya 2018). Recent computational work conducted by Jamshidi *et al*. investigating the PAβN mode of inhibition in AdeB in *A. baumannii* revealed that it occupies the hydrophobic distal binding pocket to keep the binding monomer in the binding configuration, thus preventing the pump from progressing through the series of conformational changes required to achieve substrate efflux (Jamshidi, Sutton and Rahman 2017).	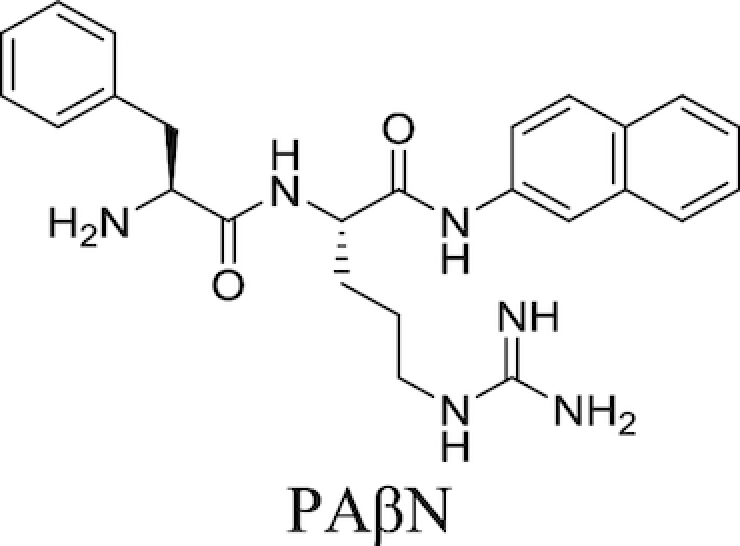
Quinoline derivatives	Quinoline compounds and their derivatives have been shown able to inhibit efflux of various antibiotics in MDR isolates of *Klebsiella aerogenes* (previously *Enterobacter aerogenes*). Compound 814 was reported to potentiate chloramphenicol 16-fold (512 µg mL^-1^ to 32 µg mL^-1^) and norfloxacin 8-fold (128 µg mL^-1^ to 16 µg mL^-1^) against the MDR strain EA3 (Mahamoud *et al*. 2006).	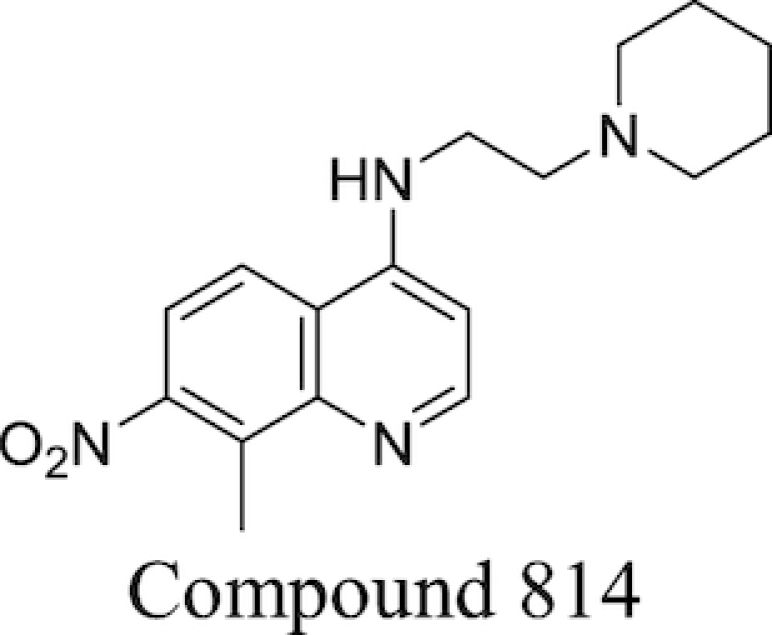
Pyridoquinoline derivatives	Pyridoquinoline derivatives have been found to restore fluoroquinolone activity in *K. aerogenes*. Compound 2a was demonstrated to potentiate both norfloxacin and ciprofloxacin eight-fold (128 µg mL^-1^ to 16 µg mL^-1^ and 32 µg mL^-1^ to 4 µg mL^-1^, respectively) against the MDR strain EA3 (Chevalier *et al*. 2001).	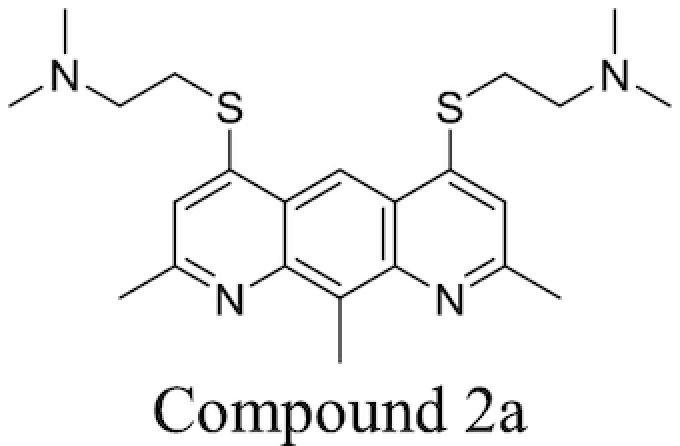
Arylpiperazine derivatives	1-(1-Naphthylmethyl)-piperazine inhibits both the AcrAB and AcrEF efflux pumps in *E. coli*, increasing levofloxacin susceptibility (among other antibacterial agents) in *E. coli* clinical isolates. It also potentiated antimicrobial activity in several Enterobacteriaceae species, including *K. pneumoniae*, *K. aerogenes*, *A. baumannii* and *Vibrio cholera* (Bohnert and Kern 2005; Pannek *et al*. 2006; Schumacher *et al*. 2006; Bina, Philippart and Bina 2009). However, because of their serotonin agonist properties, compounds in this class are considered unsuitable for use as EPIs in humans (Zechini and Versace 2009).	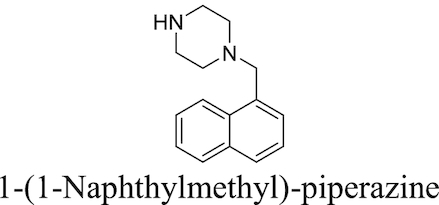
Pyridopyrimidine derivatives	Developed by Daiichi Pharmaceutical Co., lead compound D13-9001 binds to and inhibits AcrB in *E. coli* and MexB in *P. aeruginosa* by preventing the conformational changes required for the pump to successfully extrude its bound substrates (Nakashima *et al*. 2013). No clinical evaluation of D13-9001 has been published yet (Mahmood *et al*. 2016).	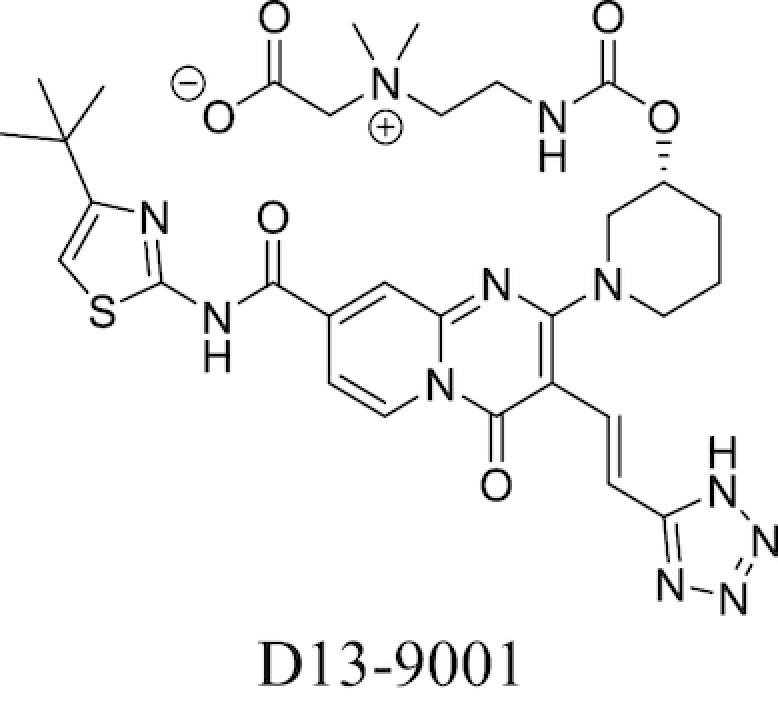
Pyranopyridine derivatives	Compound MBX-2319, found through a high-throughput screen for small molecule potentiators of ciprofloxacin in *E. coli*, has shown activity against AcrAB in *E. coli* and increases the activity of drugs that are known substrates of AcrAB (Aron and Opperman 2016). Although the compound does not show any bactericidal activity itself, it was found to cause two-, four- and eight-fold decreases in the MICs of ciprofloxacin, levofloxacin and piperacillin, respectively, when used at a concentration of 12.5 µM (Opperman *et al*. 2014; Vargiu *et al*. 2014). Based on structure-activity relationship analysis, a second generation of pyranopyridines was developed, including MBX-3796. Aron and Opperman report that MBX-3796 is ‘well tolerated at 10 mg kg^-1^ IV and [exhibits] a promising PK profile with an AUC ∼10 000 and a CL < 1000 mL hr^-1 ^kg^-1^’ (Aron and Opperman 2016). As of 2018, the current lead compound in the series is MBX-4191, and is reported to have no intrinsic antibiotic activity (MIC ≥ 100 μM), potent potentiation of antibacterials in Enterobacteriaceae but less effect in non-fermenting Gram-negatives due to poor OM penetration (Opperman 2018).	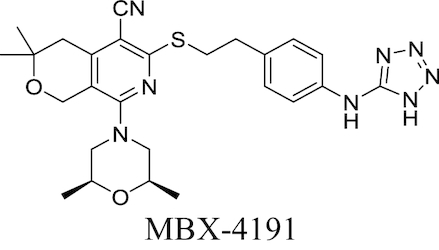
Biricodar, timcodar	Biricodar (formerly VX 710) and timcodar (formerly VX 853) were originally developed by Vertex Pharmaceuticals as anticancer agents, but have more recently found applications in prokaryotic efflux inhibition. Mullin *et al*. found both compounds capable of enhancing the activities of ethidium bromide, ciprofloxacin, tetracycline and gentamicin (amongst others) against *S. aureus* (Mullin *et al*. 2004). Further work by Grossman revealed that timcodar can synergise with the antituberculous drugs rifampicin, moxifloxacin, and bedaquiline against *M. tuberculosis* (Grossman *et al*. 2015).	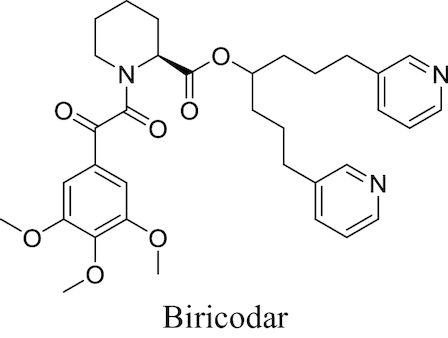
Previously-Approved Drugs		
Trimethoprim and sertraline	The combination of trimethoprim, a dihydrofolate reductase inhibitor, and sertraline, a selective serotonin reuptake inhibitor (SSRI), is synergistic with three conventional antibiotics (levofloxacin, piperacillin and meropenem) against *P. aeruginosa*. As reported by Adamson *et al*., this synergism was not present in efflux-deficient mutants of *P. aeruginosa*, indicating the efflux pump inhibitory nature of the two drugs together. Further *in vivo* evidence showed that trimethoprim and sertraline were of enhanced therapeutic benefit in *P. aeruginosa*-infected *Galleria mellonella* larvae when compared with antibiotic monotherapy (Adamson, Krikstopaityte and Coote 2015).	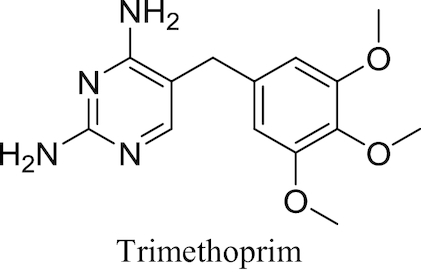
Selective serotonin reuptake inhibitors	A subclass of SSRIs termed the phenylpiperidine SSRIs (p-SSRIs), including paroxetine, were first shown to be inhibitors of the *S. aureus* MFS-type NorA pump by Kaatz and co-workers, with a group of four P-SSRIs showing consistent potentiation of both ethidium bromide (two-eight fold at 20 µg mL^-1^) and norfloxacin (four-eight fold at 20 µg mL^-1^) (Kaatz *et al*. 2003a). Subsequent structure-activity relationship work by Kaatz sought to rationalise the varying levels of potentiation achieved by the different P-SSRI analogs used (Wei, Kaatz and Kerns 2004). More recently, Nzakizwanayo and co-workers reported that the SSRI fluoxetine inhibits the *Proteus mirabilis* Bcr/CflA efflux system, determined *via* an ethidium bromide accumulation assay. Since this efflux system plays an important role in the formation of *P. mirabilis* biofilms, fluoxetine and related derivatives could prove useful as biofilm disrupting agents (Nzakizwanayo *et al*. 2017).	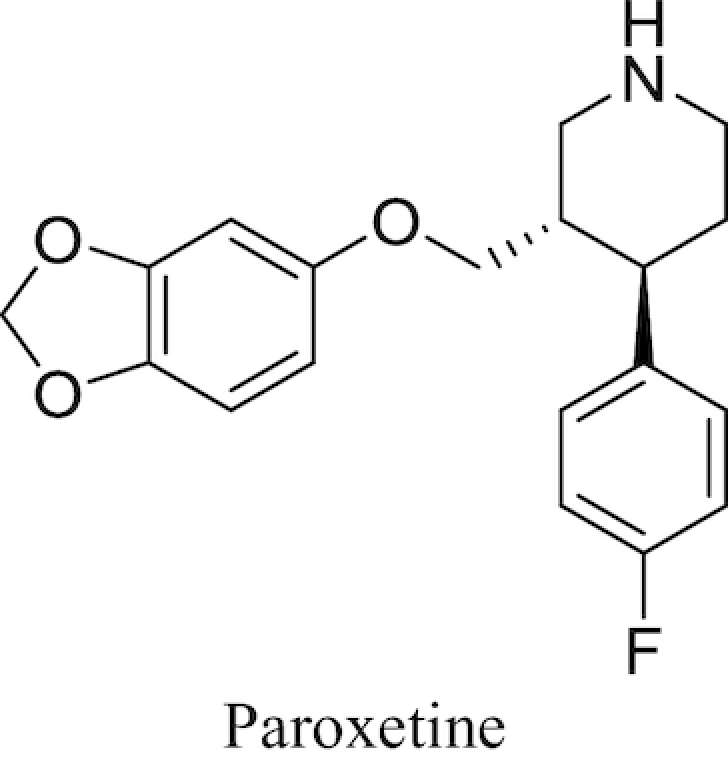
Proton pump inhibitors	Members of this class, including omeprazole and lansoprazole, have inhibitory activity towards NorA in *S. aureus*. Aeschlimann *et al*. reported eight-fold potentiation of both ciprofloxacin and norfloxacin by the aforementioned PPIs against the NorA-overexpressing *S. aureus* mutant strain SA 1199B (Aeschlimann *et al*. 1999).	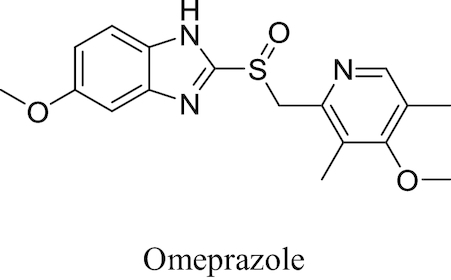
Calcium channel blockers	Verapamil, a drug used to treat cardiac disorders through inhibiting mammalian efflux transporters such as P-glycoprotein, has also been shown to inhibit the ATP-dependent ABC-type prokaryotic efflux systems. It is capable of potentiating a number of antibiotics (including rifampicin, fluoroquinolones and macrolides) against strains of *M. tuberculosis* (Pule *et al*. 2016, Chien, Yu and Hsueh 2017). The phenothiazines, including chlorpromazine and prochlorperazine, are marketed antipsychotic medications that have also been observed as a class to inhibit the MFS-type pump NorA in *S. aureus* (Kaatz *et al*. 2003b).	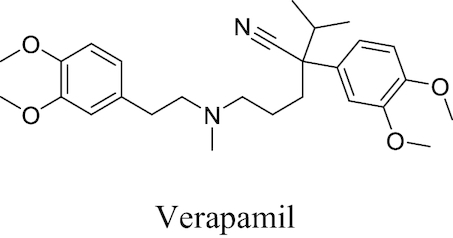

Two alternative interpretations of the EPI paradigm could address this problem. The design of agents, either small molecule or biologic in nature, capable of selectively binding the promoter regions of the genes encoding efflux transporters could allow for the efflux problem to be circumvented entirely by preventing pump expression. Jeon and Zhang employed peptide nucleic acids, synthetic DNA-mimicking polymers, to this end and achieved decreased expression of the RND-type CmeABC efflux pump in *Campylobacter jejuni*, sensitising it to ciprofloxacin and erythromycin. Addition of the peptide nucleic acid, CmeA-PNA, at a concentration of 1 µM resulted in a two-fold reduction of the MICs of both antibiotics, while at a concentration of 2 µM it caused eight- and four-fold reductions in the MICs of ciprofloxacin and erythromycin, respectively (Jeon and Zhang 2009). Alternatively, covalently modified antibiotics with EPI character could provide a conventional pump blocking agent free of the aforementioned competitive binding disadvantage and thus be better able to improve upon the parent antibiotic (Laws *et al*. 2017).

## Future perspective and conclusion

Antibiotic resistance is increasing at an alarming rate and is now widely recognised as a global issue that requires urgent attention. Despite several strategies being deployed, resistance levels are still of huge concern, and ARBs represent a promising avenue of research to counter this. Yet as things stand, the only class of ARBs to make a significant impact in the clinic is the BLIs; factors underpinning both the successes of enzyme inhibitors and the failures of EPIs and membrane permeabilisers as adjunct therapies must be appreciated if a more complete suite of clinically-approved ARBs is to be realised.

The conventional ARB approach—that of using discrete antibiotic and ARB compounds in combination to enhance the action of the former—deserves re-examining. Undoubtedly it has advantages, chiefly an inherent flexibility in the nature of the combined agents and the possibility of synergy between the two drugs. But these are offset by a number of problems, including the increased regulatory burden resulting from combining two drugs and a caveat that the pharmacokinetic profiles of the combined drugs be similar (Gonzalez-Bello 2017). The latter has suited development of β-lactam-BLI combinations since BLIs necessarily resemble β-lactam antibiotics in structure, but likely poses more of a challenge in the cases of membrane permeabilisers and EPIs where the ARBs will likely not structurally resemble the antibiotic they potentiate. This hurdle may be circumvented by embracing other methodologies, such as covalent modification of substrate antibiotics to introduce additional ARB character (Laws *et al*. 2017).

Further research within the field must aim for derivatives with improved toxicological profiles, since several of the compounds mentioned herein are unsuitable for further clinical development for this reason (Zabawa *et al*. 2016; Lomovskaya 2018). In this regard, the investigation of less nephrotoxic derivatives of polymyxin B (Corbett *et al*. 2017; Zurawski *et al*. 2017) (ClinicalTrials.gov, NCT03022175 & NCT03376529) and continued refinement of existing EPI scaffolds (Opperman *et al*. 2014, Vargiu *et al*. 2014; Aron and Opperman 2016; Opperman 2018) is encouraging. Another option here, as noted by David Brown in his 2015 review on the subject, is the repurposing of previously-approved drugs for use as ARBs or their use as hit scaffolds in ARB development (Brown 2015). This would presumably serve to expedite the market entry of any resulting therapies and is an attractive option.

*De novo* techniques must play a role in ARB development; an area which will likely drive development of future ARBs through enhancing understanding of ARB mechanisms of action is computational modelling of specific biological targets and systems. This is particularly true in the case of efflux inhibition, where in the absence of crystal structures (due to the complex, transmembrane nature of prokaryotic efflux transporters), use of computer processing power to develop a mechanistic understanding of efflux inhibition is critical (Ramaswamy *et al*. 2016; Jamshidi, Sutton and Rahman 2018). The current state of technology necessitates a compromise between accuracy and computational burden; systems on the protein scale are modelled using coarse grain molecular dynamics simulations, with more accurate and resource-intensive quantum mechanical simulations reserved only for small areas therein (Chaskar, Zoete and Rohrig 2017). However, with increasing interest and investment in much-vaunted quantum computing technology (Preskill 2018), the gains in processor power required for quantum mechanical simulations to be applied to protein-sized systems may soon be within reach. Such advances could conceivably allow a more accurate suite of *in silico* modelling tools to drive new generations of both antibiotic and ARB compounds towards the clinic.

The scientific community can also look beyond small-molecules to biologics and related technologies in order to realise the next generation of ARBs. Researchers need to further explore the use of biologics in targeted delivery to overcome resistance and reduce the selection pressure associated with non-targeting broad-spectrum antibiotics. The success of antibody-drug conjugates as cancer therapies has led to research into antibiotic-antibody conjugates using bacteria-specific antibodies (Mariathasan and Tan 2017) and there has been some early success at the pre-clinical level to treat intracellular *S. aureus* (Lehar *et al*. 2015). Phage therapy, which uses viruses that specifically infect bacterial cells, also deserves mention; though its discovery and first use predates that of modern antibiotics, doubts surrounding the efficacy of phage preparations led to their supersession by the latter (Sulakvelidze, Alavidze and Morris 2001). Phage therapy is not widely used currently and is approved in few countries (Sulakvelidze, Alavidze and Morris 2001), but previous data shows its potential for treating infections of *E. coli* (Smith and Huggins 1982), *P. aeruginosa*, *A*. *baumannii* (Soothill 1992) and *K. pneumoniae* (Bogovazova *et al*. 1991) in mice and several phage preparations have undergone phase I/II clinical trials, including a topical preparation for *E. coli* and *P. aeruginosa* infections in burn wounds (Gill, Franco and Hancock 2015).

The use of nucleic acid-based aptamers is another promising direction and can be used for the specific recognition of infectious agents as well as for blocking their functions. Systematic evolution of ligands by exponential enrichment (SELEX) technologies are being employed to identify aptamers that can detect specific pathogens (Alizadeh *et al*. 2017). Aptamers could be used to develop nucleic acid-based detection systems that can detect bacteria directly in a real complex matrix without preliminary concentration, which is often a limiting factor in developing rapid diagnostics. Aptamers able to detect and often block critical function have already been reported for *S. enterica*, *S. aureus* and *M. tuberculosis* and this represents an important development towards the realisation of such diagnostic platforms (Alizadeh *et al*. 2017).

A novel delivery platform using nanocarriers could be used to overcome the permeability barrier encountered in Gram-negative bacteria. Nanocarriers can also be used to selectively deliver high concentrations of antibiotics locally, thus avoiding systemic side effects. Several strategies have been studied in order to deliver antibiotics such as the use of antimicrobial polymers, nanoparticles and liposomes. Success with these strategies has been limited, but it is expected that with more research and advancement of technology, nanodelivery can become an important tool to overcome bacterial resistance (Gao *et al*. 2014).

The BLIs in the clinic today were, by their very nature, developed after their partner antibiotics. However, the fact that the majority of BLIs have arrived on the clinical scene many decades after their partner β-lactams were first approved (Drawz and Bonomo 2010) likely reflects the relatively recent drive to address the problem of AMR. This typifies the current reactive nature of antibiotic research and development, ‘patching up’ a failing arsenal as it declines. Going forward, we hope that a new wave of funding schemes such as non-profit private-public partnerships (such as CARB-X) and government-funded programmes (such as the EU-backed Innovative Medicines Initiative) can drive a change in this methodology. A more proactive, diagnostics-driven approach to ARB development would allow the lifespans of current antibiotics to be maximised when practised in combination with wider efforts such as antimicrobial stewardship and increased public awareness of AMR.

## Notes

All authors researched data for the article, made substantial contributions to discussions of the content, wrote the article and reviewed and edited the manuscript before submission.
